# Elucidating Novel Serum Biomarkers Associated with Pulmonary Tuberculosis Treatment

**DOI:** 10.1371/journal.pone.0061002

**Published:** 2013-04-18

**Authors:** Mary A. De Groote, Payam Nahid, Leah Jarlsberg, John L. Johnson, Marc Weiner, Grace Muzanyi, Nebojsa Janjic, David G. Sterling, Urs A. Ochsner

**Affiliations:** 1 SomaLogic, Inc., Boulder, Colorado, United States of America; 2 Department of Microbiology, Immunology and Pathology, Colorado State University Campus, Fort Collins, Colorado, United States of America; 3 Pulmonary and Critical Care Medicine, University of California San Francisco, San Francisco, California, United States of America; 4 Tuberculosis Research Unit, Division of Infectious Diseases, Case Western Reserve University, Cleveland, Ohio, United States of America; 5 Division of Infectious Diseases, University of Texas Health Science Center, San Antonio, Texas, United States of America; 6 Uganda-Case Western Reserve University Research Collaboration, Kampala, Uganda; Oxford University, United Kingdom

## Abstract

In an unbiased approach to biomarker discovery, we applied a highly multiplexed proteomic technology (SOMAscan, SomaLogic, Inc, Boulder, CO) to understand changes in proteins from paired serum samples at enrollment and after 8 weeks of TB treatment from 39 patients with pulmonary TB from Kampala, Uganda enrolled in the Center for Disease Control and Prevention’s Tuberculosis Trials Consortium (TBTC) Study 29. This work represents the first large-scale proteomic analysis employing modified DNA aptamers in a study of active tuberculosis (TB). We identified multiple proteins that exhibit significant expression differences during the intensive phase of TB therapy. There was enrichment for proteins in conserved networks of biological processes and function including antimicrobial defense, tissue healing and remodeling, acute phase response, pattern recognition, protease/anti-proteases, complement and coagulation cascade, apoptosis, immunity and inflammation pathways. Members of cytokine pathways such as interferon-gamma, while present, were not as highly represented as might have been predicted. The top proteins that changed between baseline and 8 weeks of therapy were TSP4, TIMP-2, SEPR, MRC-2, Antithrombin III, SAA, CRP, NPS-PLA2, LEAP-1, and LBP. The novel proteins elucidated in this work may provide new insights for understanding TB disease, its treatment and subsequent healing processes that occur in response to effective therapy.

## Introduction

Using an unbiased approach with a highly multiplexed comprehensive platform, our goal in this study was to identify and quantify protein markers that are present in patients diagnosed with culture-confirmed active TB and that change in response to highly efficacious drug therapy.

In our previous work in lung cancer we performed a large scale application of our platform technology to identify markers capable of diagnosing early onset lung cancer [1]. In this study we used the same modified aptamer array to quantitate 1,030 proteins in serum of patients diagnosed with active TB disease and monitor changes in all markers to better understand the evolution of protein markers as the disease improves on anti-mycobacterial therapy. SOMAscan proteomic technology is based on slow off-rate modified aptamers (SOMAmers), with improved binding properties due to long dissociation rates (generally >30 min) and the incorporation of modified nucleotides that lead to higher affinity of these reagents as compared to standard RNA or DNA aptamers. SOMAmers are made from single-stranded DNA (ssDNA) that contain pyrimidine residues modified at their 5-prime position to introduce functional groups not present in natural nucleic acids, such as mimics of amino acid side-chains. SOMAmers have several advantages over antibodies, including lower molecular weight, higher multiplexing capabilities (low cross-reactivity, universally-applicable assay conditions), chemical stability (to heat, drying, and solvents, reversible renaturation), ease of reagent manufacturing, consistent lot-to-lot performance and lower cost (fully synthetic).

The Version 2 SOMAscan assay generates simultaneous quantitative measurements of 1,030 human proteins in serum, plasma, CSF or tissue lysate [2] in a small (<100 µl) sample volume. Across all 1,030 proteins, the median lower limit of quantitation is 0.3 picomolar (pM), with a dynamic range of >5 logs, and a median coefficient of variation (%CV) of 5% [3].

This proof-of-principle study is the first large-scale unbiased targeted proteomic analysis employing modified DNA aptamers and we sought to identify and quantify protein markers that are associated with active TB and that changed in response to four-drug treatment. This pilot study was made possible due to an archived collection of specimens nested into Tuberculosis Trials Consortium (TBTC) Study 29, a phase 2B clinical trial which evaluated rifapentine in place of rifampin in combination with isoniazid, ethambutol and pyrazinamide for the treatment of drug-susceptible TB [4].

## Results

### Participant Characteristics

Clinical characteristics of the 39 participants included in this study are listed in Table 1. Participants completed between 6 and 24 months of anti-TB treatment. Follow-up through the end of treatment did not reveal any treatment failures. One participant had INH and RIF resistant tuberculosis; one participant had mono-drug resistance to INH and one had dual resistance (to streptomycin and rifampin), all were detected after the participants completed intensive phase treatment. Four participants received between 3–5 days of 4 drug, standard chemotherapy prior to enrollment.

**Table 1:** TB patient characteristics.

Patient characteristics	Value (range)
# patients	39
Rifampin treated (%)	14 (36)
Age years, median	28.5 (19–53)
Male (%)	28 (72)
BMI median, kg/m^2^	19 (15.2–26.7)
Cavitary lesions (%)	56
Smoker (%)	20
Alcohol use (%)	2

### Sample Handling and Analysis

A small systematic difference (4%) in the overall protein concentrations between the baseline and 8 week sample sets was removed during normalization. Three samples had elevated hemoglobin levels and correspondingly low haptoglobin levels (data not shown) when compared both to other subjects and internal assay calibrators suggesting some degree of hemolysis. No other evidence of sample processing errors [3] was observed so all samples were considered fit for inclusion in the subsequent data analysis.

### Nonspecific Markers of Active TB

**Figure 1:**
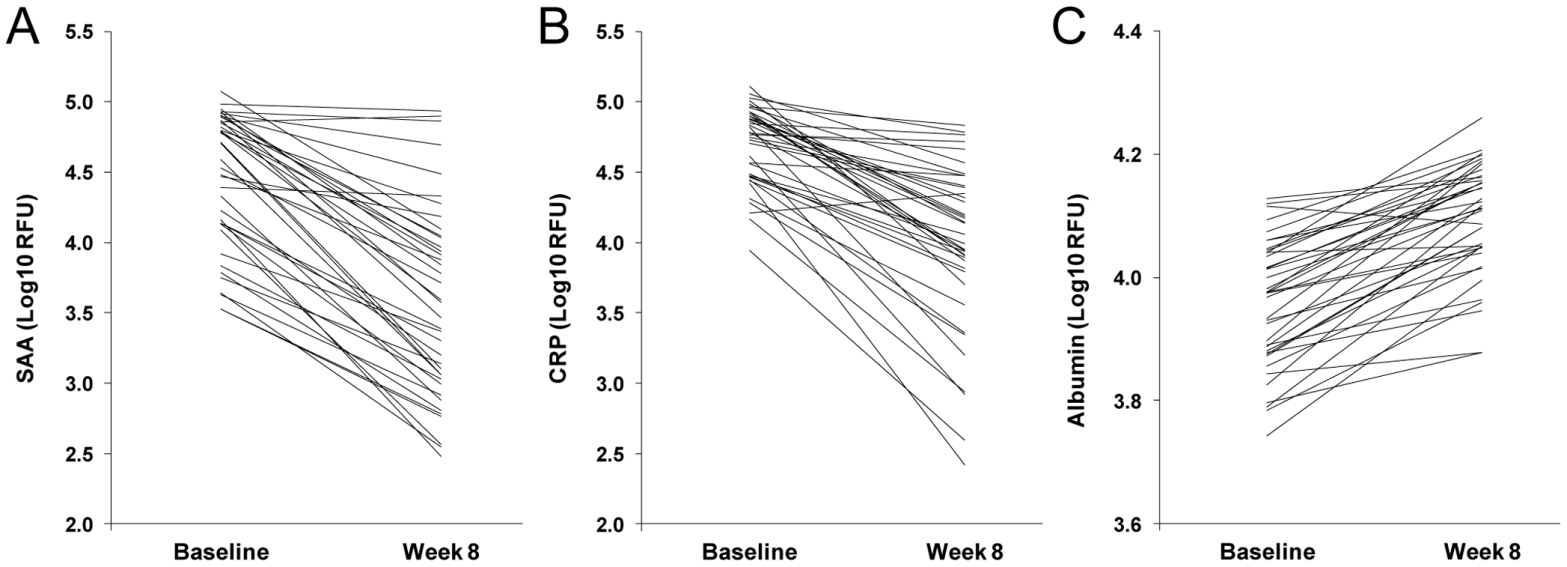
Changes in expression of non-specific markers for active TB, including acute phase reactants SAA (A) and CRP (B), and albumin (C), between baseline and week 8 of therapy.

Serum protein concentrations in TB patients at baseline were compared to those measured in the same patients after 8 weeks of therapy. The acute phase reactants C-reactive protein (CRP) and serum amyloid A protein (SAA) decreased from baseline to week 8 in all but one subject (Figure 1). Serum albumin increased between baseline and week 8 in all but one subject. Other known important acute phase reactants including haptoglobin, alpha-1 antitrypsin (AAT) and serum amyloid A protein declined from baseline to week 8 consistent with a reduction in the disease burden.

### Correlations with Severity of Disease

**Figure 2:**
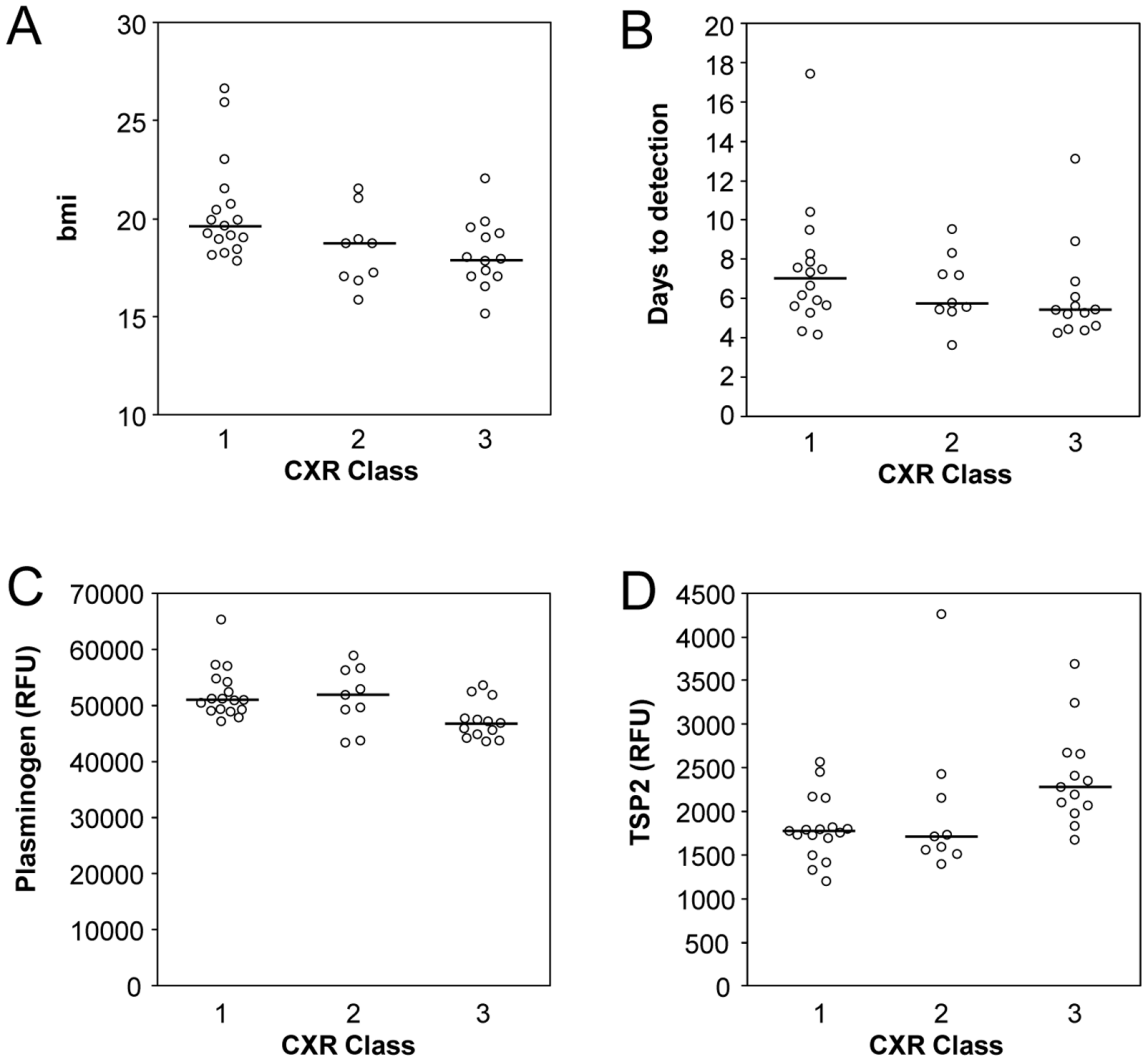
Association of clinical parameters (BMI, time to detection) and serum protein levels (plasminogen, thrombospondin-2) with radiographic classification of cavitation.

Microbiological and radiographic parameters are used to assess the severity of TB disease. At baseline, total cavitary volume and time-to-detection in MGIT culture are markers of severity of disease and bacillary burden in the sputum, respectively. Among the data available for the patients involved in our study, CXR class (as defined by presence cavitation and size on baseline CXR, class 1 = no cavitation; class 2 = cavitation present, <4 cm in size; class 3 = cavitation present, >4 cm in size) showed the strongest association with other parameters such as body mass index (BMI, Figure 2A) and time-to-detection in MGIT culture (TTD, Figure 2B), and as expected, both were lower in the 13 patients with large and/or bilateral cavities (CXR class 3) compared to the 17 patients without cavitary disease (CXR class 1), but there was substantial overlap between groups. Plasminogen (Figure 2C), which was lower in CXR class 3 compared to class 1 and thrombospondin-2 (TSP-2), which was higher in CXR class 3 compared to class 1 (Figure 2D) best discriminate CXR class 1 from class 3 patients, though these effects had an 18% false discovery rate, so on average we expect roughly 1 in 6 such effects to be “false” discoveries.

The top markers at baseline distinguishing the thirteen patients with more severe disease (score >0.60) and the thirteen patients with less severe disease (score <0.40) were CRP, SAA, and NPS-PLA2 with roughly two-fold increased levels of the median.

**Figure 3:**
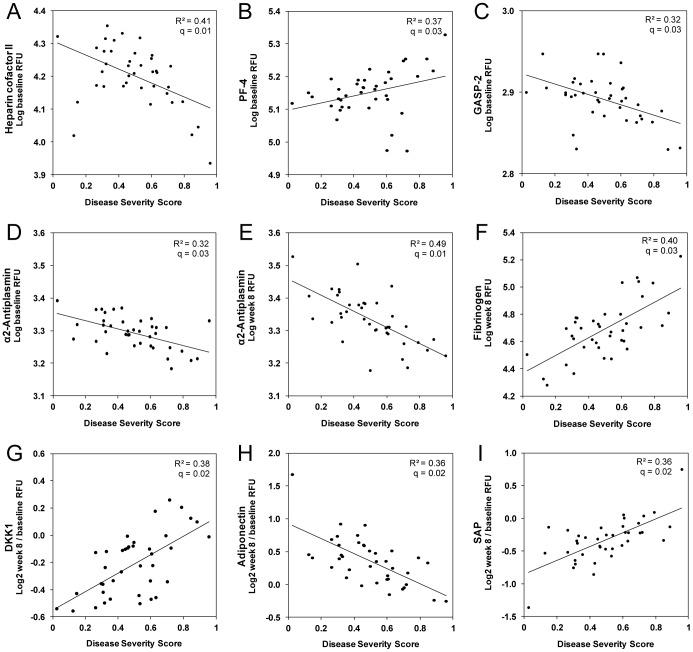
Correlation of serum protein markers with TB disease severity. Top markers that showed the largest differential expression in mild disease (*n* = 13) compared to severe disease (*n* = 13), based on medians at baseline. Log10 RFU was used as the response in a linear model and a 5% false discovery rate, A–D: Markers of disease severity identified via linear regression analysis of the protein concentration correlated with disease severity score at baseline. E-F: Levels of α2-antiplasmin and fibrinogen (respectively) relative to disease severity score at 8 weeks. G-I: Markers of disease severity based on a regression analysis of the expression shift from baseline to week 8.

Regression analysis was used to identify proteins that changed with increasing disease severity as measured by our custom, composite score. Using baseline (log) RFU as the response in a linear model and a 5% false discovery rate, Heparin cofactor 2, platelet factor-4 (PF-4), G-protein coupled receptor associated sorting protein-2 (GASP-2), and α2-antiplasmin had baseline concentrations that were correlated with our disease severity score (Figure 3A–D). Low levels of heparin cofactor 2 and GASP2 were both associated with more severe disease. Interestingly, α2-antiplasmin levels at 8 weeks also decreased with increasing severity scores (Figure 3E). In contrast, fibrinogen levels measured at 8 weeks were higher in patients with severe disease (Figure 3F). Regression analysis using the (log 2) ratio of week 8 to baseline concentration and a 5% false discovery rate uncovered additional proteins whose rate of change was associated with disease severity. The top markers were DKK-1, adiponectin and serum amyloid P component (SAP) (Figure 3G–I). DKK-1 levels decreased from baseline to 8 weeks in patients with mild disease, but remained high or even increased in patients with more severe disease. Adiponectin increased in the majority of patients with mild disease but remained unchanged in those with the highest disease severity score (data not shown).

### Paired Analysis of Serum Proteins at Baseline versus Week 8 of TB Therapy

A large number of proteins had levels that changed between baseline and 8 weeks of treatment. At a 0.01% false discovery rate (q <0.0001), 239 of the 1,030 proteins measured were differentially expressed between the two time points. Sixteen proteins shifted in the same direction from baseline to week 8 in all 39 patients (11 increased and 5 decreased from baseline), and many other proteins showed a consistent shift from baseline to week 8 in at least ¾ (30 of 39) of the patients (Table 2). The top intra-subject markers (q<10^−6^) are shown in Table 2 (for the list of proteins with a 0.01% false discovery rate (q<10^−4^) see Table S3).

**Table 2:** Differential protein expression between baseline and week 8 in paired samples from *n* = 39 patients treated for pulmonary TB.

Rank	Target	Swiss Prot*	Intrasubject Shift (baseline to week 8)		p-value	q-value
			Up (*n*)	Down (*n*)		
1)	TIMP-2	P16035	39		5.3e-08	5.48e-07
2)	GFRα-2	O00451	39		5.3e-08	5.48e-07
3)	MRC2	Q9UBG0	39		5.3e-08	5.48e-07
4)	Haptoglobin, Mixed Type	P00738		39	5.3e-08	5.48e-07
5)	LBP	P18428		39	5.3e-08	5.48e-07
6)	amyloid precursor protein	P05067		39	5.3e-08	5.48e-07
7)	BGH3	Q15582	39		5.3e-08	5.48e-07
8)	TSP4	P35443	39		5.3e-08	5.48e-07
9)	FETUB	Q9UGM5	39		5.3e-08	5.48e-07
10)	PCI	P05154	39		5.3e-08	5.48e-07
11)	Kallistatin	P29622	39		5.3e-08	5.48e-07
12)	α2-HS-Glycoprotein	P02765	39		5.3e-08	5.48e-07
13)	CHL1	O00533	39		5.3e-08	5.48e-07
14)	CDON	Q4KMG0	39		5.3e-08	5.48e-07
15)	D-dimer	P02671, P02675, P02679		39	5.3e-08	5.48e-07
16)	MMP-1	P03956		39	5.3e-08	5.48e-07
17)	contactin-1	Q12860	38		5.7e-08	5.48e-07
18)	CD109	Q6YHK3	38		5.7e-08	5.48e-07
19)	IGFBP-7	Q16270	38		5.7e-08	5.48e-07
20)	Sphingosine kinase 1	Q9NYA1		38	5.7e-08	5.48e-07
21)	CRP	P02741		38	5.7e-08	5.48e-07
22)	SEPR	Q12884	38		5.7e-08	5.48e-07
23)	TIMP-3	P35625		38	6.1e-08	5.48e-07
24)	Lipocalin 2	P80188		38	6.1e-08	5.48e-07
25)	NAP-2	P02775		38	6.1e-08	5.48e-07
26)	Nectin-like protein 2	Q9BY67	38		6.1e-08	5.48e-07
27)	Proteinase-3	P24158		38	6.1e-08	5.48e-07
28)	PDGF-BB	P01127		38	6.1e-08	5.48e-07
29)	MMP-2	P08253	38		6.1e-08	5.48e-07
30)	TIMP-1	P01033		37	6.6e-08	5.48e-07
31)	ROR1	Q01973	37		6.6e-08	5.48e-07
32)	IGFBP-6	P24592	37		6.6e-08	5.48e-07
33)	PAI-1	P05121		38	6.6e-08	5.48e-07
34)	Protein C	P04070	37		6.6e-08	5.48e-07
35)	C9	P02748		38	6.6e-08	5.48e-07
36)	GDF-9	O60383		37	6.6e-08	5.48e-07
37)	Carbonic anhydrase 6	P23280	37		6.6e-08	5.48e-07
38)	RBP	P02753	37		6.6e-08	5.48e-07
39)	Albumin	P02768	38		6.6e-08	5.48e-07
40)	Fibronectin	P02751	37		6.6e-08	5.48e-07
41)	Antithrombin III	P01008	38		7.2e-08	5.48e-07
42)	a1-Antitrypsin	P01009		37	7.2e-08	5.48e-07
43)	HRG	P04196	38		7.2e-08	5.48e-07
44)	Angiopoietin-1	Q15389		38	7.8e-08	5.48e-07
45)	ATS13	Q76LX8	37		7.8e-08	5.48e-07
46)	Coagulation Factor VII	P08709	37		7.8e-08	5.48e-07
47)	Afamin	P43652	38		7.8e-08	5.48e-07
48)	TrkB	Q16620	37		7.8e-08	5.48e-07
49)	GOT1	P17174		38	7.8e-08	5.48e-07
50)	Azurocidin	P20160		38	8.4e-08	5.80e-07
51)	NCAM-L1	P32004	38		9.1e-08	6.03e-07
52)	PLXC1	O60486	36		9.1e-08	6.03e-07
53)	I-TAC	O14625		38	1.1e-07	6.41e-07
54)	CYTF	O76096		37	1.1e-07	6.41e-07
55)	BPI	P17213		36	1.1e-07	6.41e-07
56)	HNRPQ	O60506		36	1.1e-07	6.41e-07
57)	PHI	P06744		36	1.1e-07	6.41e-07
58)	Cathepsin G	P08311		36	1.1e-07	6.47e-07
59)	Osteoblast-specific transcription factor 2	Q13950	37		1.1e-07	6.47e-07
60)	SAA	P02735		38	1.1e-07	6.47e-07
61)	TXD12	O95881		36	1.1e-07	6.47e-07
62)	gp130, soluble	P40189	36		1.2e-07	6.87e-07
63)	ITI heavy chain H4	Q14624		36	1.3e-07	7.30e-07
64)	CDK8/cyclin C	P49336, P24863		36	1.4e-07	7.63e-07
65)	VEGF121	P15692		38	1.4e-07	7.63e-07
66)	LRIG3	Q6UXM1	36		1.5e-07	7.98e-07
67)	MAPK14	Q16539		36	1.5e-07	7.98e-07
68)	PGRP-S	O75594		36	1.7e-07	8.24e-07
69)	RGM-C	Q6ZVN8	36		1.7e-07	8.24e-07
70)	Fibrinogen g-chain dimer	P02679		37	1.7e-07	8.24e-07
71)	MMP-9	P14780		36	1.8e-07	8.52e-07
72)	Thyroxine-Binding Globulin	P05543	35		1.8e-07	8.52e-07
73)	Cadherin-5	P33151	35		1.8e-07	8.52e-07
74)	NPS-PLA2	P14555		37	1.9e-07	9.07e-07
75)	NAP-2	P02775		36	2.1e-07	9.51e-07
76)	FN1.3	P02751	34		2.1e-07	9.51e-07
77)	Protease nexin I	P07093		38	2.3e-07	9.99e-07
78)	Plasminogen	P00747	34		2.3e-07	9.99e-07

At a false discovery rate of 10^−6^, 78 of 1,030 proteins were identified as differentially expressed using the Wilcoxon Signed Rank test (for all 239 proteins with q <10^−4^ see Table S3). The intra-subject shifts indicate the number of patients showing up or down-regulation. Also shown are the raw p-value and resulting FDR corrected “q-value”.

* Swiss Prot is a large protein sequence database widely used for protein resources.

**Figure 4:**
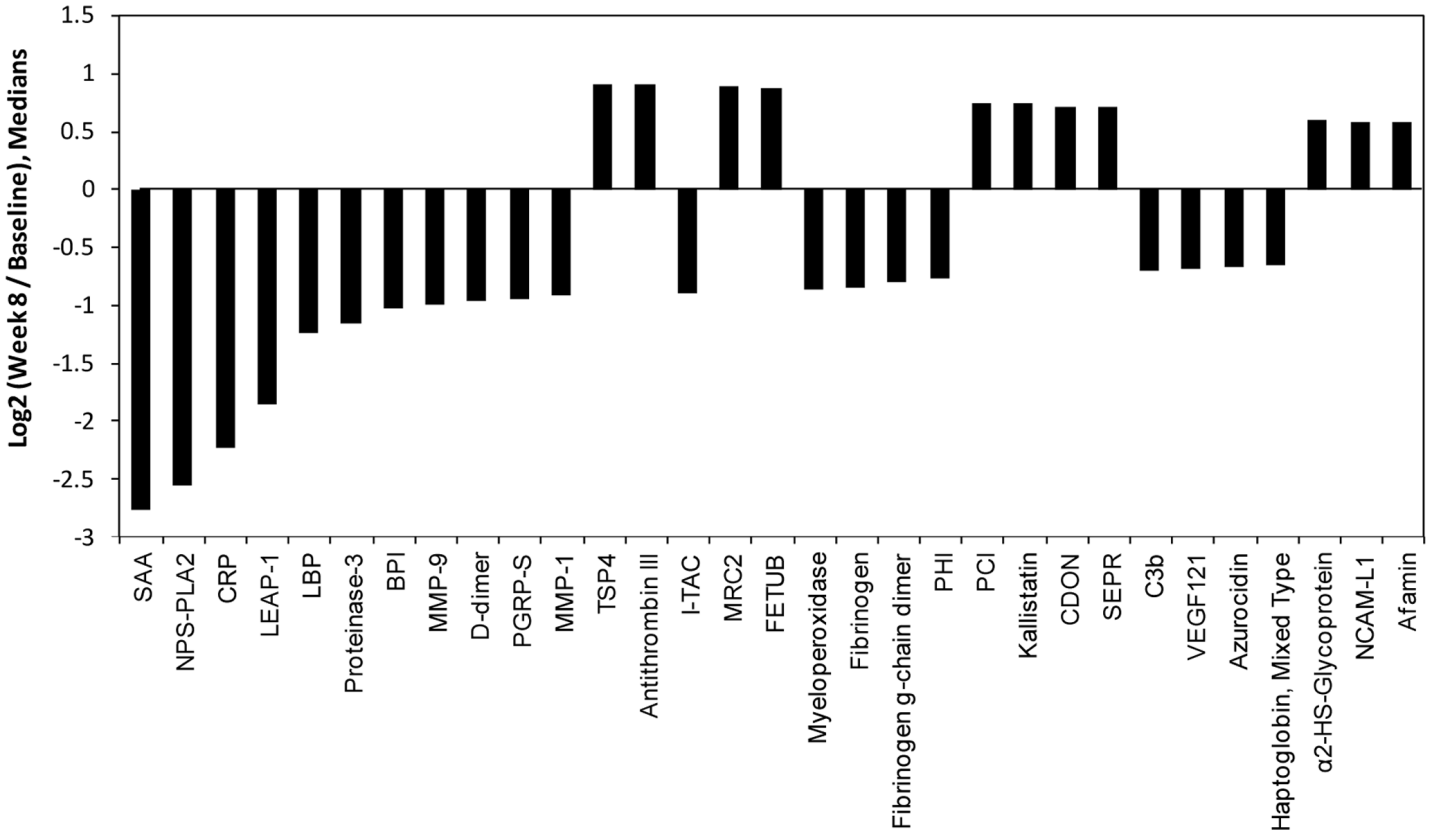
Paired analysis of markers with largest change between baseline and week 8 of TB therapy in *n* = 39 patients, ranked by the median intra-subject fold-change.

Since the signed rank test is based on the *ranks* of the measurements it is difficult to appreciate the magnitude of the changes for some of these proteins, so in Figure 4 we show the top ranked proteins using the median fold-change between baseline and week 8. SAA (Serum Amyloid A Protein), NPS-PLA2 (Phospholipase A2), and CRP (C-reactive Protein) showed median within-subject decreases of 6.8-fold, 5.9-fold, and 4.7-fold, respectively, from baseline to week 8. Sixteen additional proteins dropped at least 1.5-fold (median fold change), and a few markers increased between baseline and week 8, including TSP4, antithrombin III, mannose receptor 2 (MRC-2), fetuin-like protein (FETUB) and plasma serine protease inhibitor (PCI). We next explored the paired data using the database for annotation, visualization and integrated discovery (DAVID). Clusters based on functional annotation with an enrichment score exceeding 1.3 (corresponding to a Bonferroni corrected p-value of 0.05) were considered significant. Pathways involved in response to wounding, inflammatory response, defense response and coagulation were highly enriched. A full list of annotated clusters can be found in Figure S2.

### Unpaired Analysis

**Figure 5:**
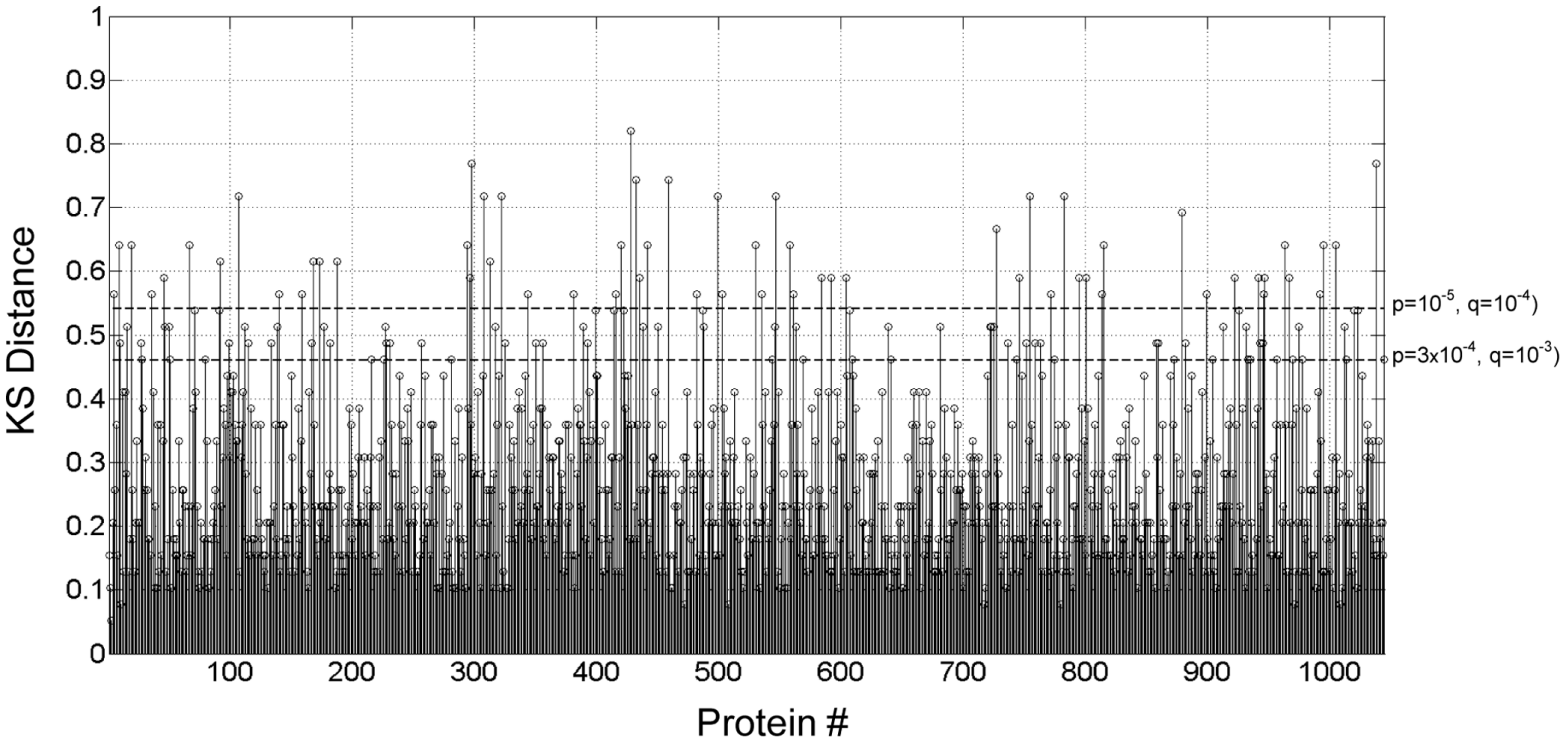
Feature separation by KS distance for 1,030 measured proteins, with corresponding significance levels shown as q-values.

**Figure 6:**
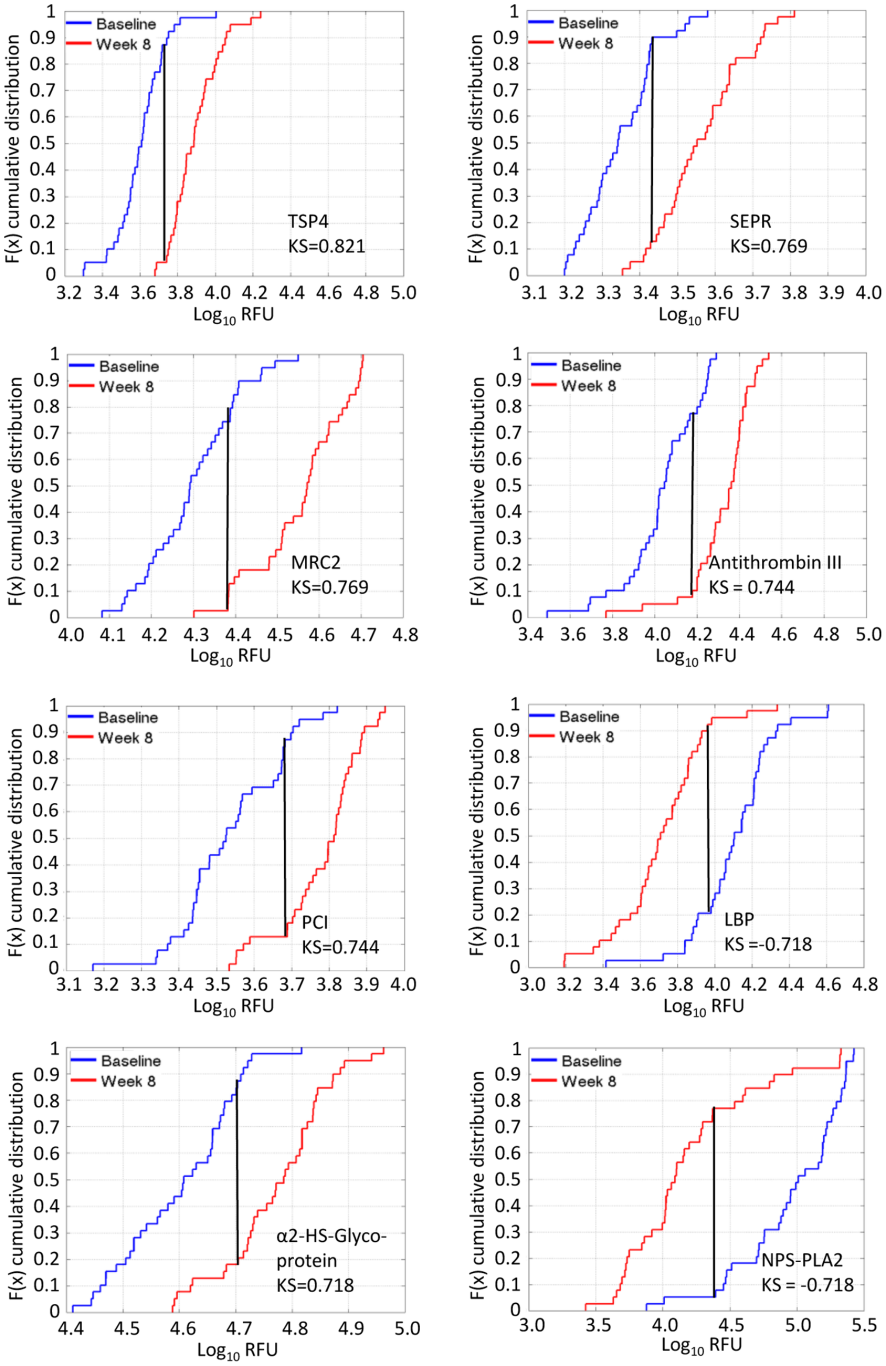
Empirical cumulative distribution functions showing Log RFU for the eight proteins with largest KS distances between baseline and week 8.

**Figure 7:**
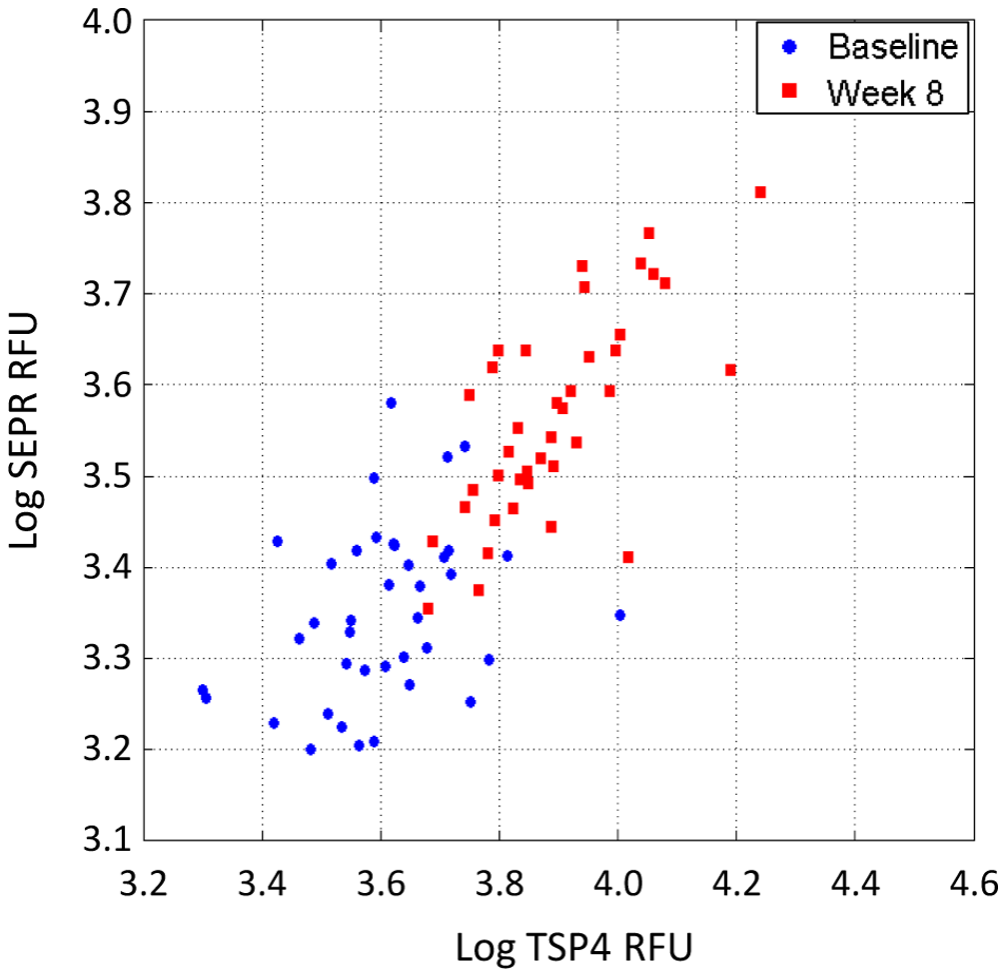
Scatter plot of the separation of baseline and week 8 samples using the two markers with the largest KS distances.

For prognostic applications we would hope to predict treatment response using only a *single* sample and with this in mind we performed an unpaired analysis to identify proteins with baseline measurement distributions that differed from the week 8 distributions. At a 0.1% false discovery rate (q <0.001) the KS test identified 116 of 1,030 proteins as differentially expressed between baseline and week 8. KS distances for all proteins are shown in Figure 5. The top 60 markers (q <10^−4^) are listed in Table 3 and all 116 markers (q<0.001) are listed in Table S4. A total of 55/116 features were up-regulated and 61/116 were down-regulated over time. The most significant changes were noted for TSP4, fibroblast activation protein α (SEPR), MRC-2, antithrombin III, PCI, LPS-binding protein (LBP), α2-HS-glycoprotein, and phospholipase A2 (NPS-PLA2). The empirical cumulative distribution functions for the baseline and week-8 protein measurements (along with an indication of the KS distance between the distributions) for the top eight markers are shown in Figure 6, and the plots for 59 additional proteins are available in Figure S3. A scatter plot using the top two markers that were differentially expressed between baseline and 8 week samples can be seen in Figure 7. Using measurements of one or more of these proteins it is possible classify each sample within this data set as belonging to either the baseline group (TB) or the week 8 group (treated TB) with sensitivity and specificity exceeding 90% (data not shown).

**Table 3:** Unpaired analysis of proteins showing differential expression between baseline and week 8 in *n* = 39 TB patients.

Rank	Target	Swiss Prot*	KS-distance (Signed)	p-value	q-value
1)	TSP4	P35443	0.821	1.37e-12	1.06e-09
2)	SEPR	Q12884	0.769	4.08e-11	1.05e-08
3)	MRC2	Q9UBG0	0.769	4.08e-11	1.05e-08
4)	Antithrombin III	P01008	0.744	2.05e-10	3.15e-08
5)	PCI	P05154	0.744	2.05e-10	3.15e-08
6)	LBP	P18428	−0.718	9.73e-10	6.25e-08
7)	α2-HS-Glycoprotein	P02765	0.718	9.73e-10	6.25e-08
8)	NPS-PLA2	P14555	−0.718	9.73e-10	6.25e-08
9)	Haptoglobin, Mixed Type	P00738	−0.718	9.73e-10	6.25e-08
10)	Kallistatin	P29622	0.718	9.73e-10	6.25e-08
11)	MMP-2	P08253	0.718	9.73e-10	6.25e-08
12)	NCAM-L1	P32004	0.718	9.73e-10	6.25e-08
13)	CDON	Q4KMG0	0.692	4.38e-09	2.60e-07
14)	Fibronectin	P02751	0.667	1.87e-08	1.03e-06
15)	Cathepsin G	P08311	−0.641	7.53e-08	2.23e-06
16)	gp130, soluble	P40189	0.641	7.53e-08	2.23e-06
17)	Nectin-like protein 2	Q9BY67	0.641	7.53e-08	2.23e-06
18)	LEAP-1	P81172	−0.641	7.53e-08	2.23e-06
19)	CRP	P02741	−0.641	7.53e-08	2.23e-06
20)	Fibrinogen g-chain dimer	P02679	−0.641	7.53e-08	2.23e-06
21)	TIMP-2	P16035	0.641	7.53e-08	2.23e-06
22)	IL-19	Q9UHD0	0.641	7.53e-08	2.23e-06
23)	CDK8/cyclin C	P49336, P24863	−0.641	7.53e-08	2.23e-06
24)	CHL1	O00533	0.641	7.53e-08	2.23e-06
25)	D-dimer	P02671, P02675, P02679	−0.641	7.53e-08	2.23e-06
26)	CATZ	Q9UBR2	0.641	7.53e-08	2.23e-06
27)	TrkC	Q16288	0.615	2.88e-07	7.15e-06
28)	Fibrinogen	P02671, P02675, P02679	−0.615	2.88e-07	7.15e-06
29)	Angiopoietin-1	Q15389	−0.615	2.88e-07	7.15e-06
30)	Lipocalin 2	P80188	−0.615	2.88e-07	7.15e-06
31)	C9	P02748	−0.615	2.88e-07	7.15e-06
32)	MMP-9	P14780	−0.590	1.04e-06	1.82e-05
33)	I-TAC	O14625	−0.590	1.04e-06	1.82e-05
34)	BMP-1	P13497	0.590	1.04e-06	1.82e-05
35)	BMPER	Q8N8U9	0.590	1.04e-06	1.82e-05
36)	Plasminogen	P00747	0.590	1.04e-06	1.82e-05
37)	PHI	P06744	−0.590	1.04e-06	1.82e-05
38)	TrkB	Q16620	0.590	1.04e-06	1.82e-05
39)	Coagulation Factor IX	P00740	−0.590	1.04e-06	1.82e-05
40)	GOT1	P17174	−0.590	1.04e-06	1.82e-05
41)	RBP	P02753	0.590	1.04e-06	1.82e-05
42)	Albumin	P02768	0.590	1.04e-06	1.82e-05
43)	Sphingosine kinase 1	Q9NYA1	−0.590	1.04e-06	1.82e-05
44)	Afamin	P43652	0.590	1.04e-06	1.82e-05
45)	TIMP-1	P01033	−0.564	3.56e-06	4.58e-05
46)	GFRa-2	O00451	0.564	3.56e-06	4.58e-05
47)	Azurocidin	P20160	−0.564	3.56e-06	4.58e-05
48)	Lactoferrin	P02788	−0.564	3.56e-06	4.58e-05
49)	amyloid precursor protein	P05067	−0.564	3.56e-06	4.58e-05
50)	RET	P07949	0.564	3.56e-06	4.58e-05
51)	LRIG3	Q6UXM1	0.564	3.56e-06	4.58e-05
52)	CD30 Ligand	P32971	0.564	3.56e-06	4.58e-05
53)	Osteoblast-specific transcription factor 2	Q13950	0.564	3.56e-06	4.58e-05
54)	Proteinase-3	P24158	−0.564	3.56e-06	4.58e-05
55)	MASP3	P48740	0.564	3.56e-06	4.58e-05
56)	HNRPQ	O60506	−0.564	3.56e-06	4.58e-05
57)	SAA	P02735	−0.564	3.56e-06	4.58e-05
58)	PLXC1	O60486	0.564	3.56e-06	4.58e-05
59)	Coagulation Factor IX	P00740	−0.564	3.56e-06	4.58e-05
60)	CAPG	P40121	−0.564	3.56e-06	4.58e-05

A total of 60 proteins were differentially expressed using the KS test and 0.01% false discovery rate (q <10^−4^; for all 116 proteins with q <10^−3^ see Table S4). The shift in expression is shown as the signed KS-distance, with a positive KS value indicating up-regulation of the protein after 8 weeks of TB therapy. Also shown are the raw p-value and resulting FDR corrected “q-value”.

* Swiss Prot is a large protein sequence database widely used for protein resources.

## Discussion

In this study, we conducted unbiased targeted proteomic analysis employing modified DNA aptamers to identify and quantify protein markers that were associated with active TB and that changed in response to four-drug treatment. To date, we have conducted several other blood-based clinical biomarker studies of human diseases, including lung cancer [3], West Nile Virus infection (unpublished) and chronic kidney disease [2]. These studies have identified novel disease biomarkers as well as confirmed associations of previously reported biomarkers. Recent biomarker discovery programs aimed at identifying novel TB biomarkers with demonstrable utility have been successful [17]–[19]. Among the analytical tools for TB biomarker discovery, transcriptional microarray [17], [20], [21], 2D gel analysis [22], and mass spectroscopy [18], [23] have been used but none, to our knowledge, has undertaken such a large-scale analysis for the targeted detection of protein levels in the serum of patients with TB disease.

Increasing serum albumin levels and declining levels of acute phase reactant proteins over time gives confidence in our findings as it is consistent with the anticipated improvement of TB patients in response to combination drug therapy. We observed a lack of change in classically described soluble cytokines (e.g. interferon-γ, tumor necrosis factor-α, interleukin-1 β, etc.) which have been implicated in TB pathogenesis [20], [21], [24]–[26] and as treatment response biomarkers [24]. The healing process that accompanies effective anti-TB therapy appears to be highly associated with a fibrotic healing process and is in keeping with radiographic changes known to occur with TB therapy. Among the diverse pathways we identified, the most predominant were those representing the core biological themes of antimicrobial defenses and tissue remodeling/healing functions.

### Biomarkers of Inflammation and Anti-microbial Defense

We identified differentially expressed proteins involved in innate and adaptive immunity to which antimicrobial function has been attributed, including but not limited to; complement cascade components, CRP [27], α-1 antitrypsin (AAT) [28], [29], hepcidin (LEAP) [30], bactericidal permeability increasing protein (BPI) [31], lipopolysaccharide binding protein (LBP) [32] and phospholipase A2 (NPS-PLA2) [33] which all decreased over time in the majority of patients. C9 and C3 breakdown products (C3b and C3d) decreased on therapy in the majority of the patients. Components of the MTB bacillus are known to induce the antimicrobial molecule hepcidin (LEAP) which has been reported to have both iron handling and antibacterial properties [30]. The finding of NPS-PLA2 as a top marker distinguishing week 8 samples from baseline samples highlights the importance of this protein which has both antibacterial and lipolytic functions in the host. NPS-PLA2 is an innate immune antibacterial molecule involved in arachidonic acid and fatty acid generation and may be involved in the lipoid pneumonia seen with pulmonary TB [34]. We have discovered several host enzymes involved in lipid metabolism (a sphingosine kinase and a phospholipase); the significance of these discoveries is unknown, but may relate to important bioactive lipid metabolites in TB. Cathepsin G, an antimicrobial molecule and serine protease found in hypoxic TB granulomas [35] decreased on therapy in the majority of patients. Mannose receptor C type 2 (MRC-2), another pattern recognition molecule known to be involved in TB and activating the innate immune system [36] was among the top 3 markers that were differentially expressed between baseline and 8 weeks.

### Biomarkers of Tissue Remodeling

In this work, we discovered many proteins involved in tissue healing including proteases and anti-proteases, fibrotic process proteins, remodeling of collagen and extracellular matrix (ECM) as well as members of the coagulation cascade. Plasminogen, a marker found in all analyses has been reported to be co-opted by MTB [37] and other respiratory pathogens [38] in order to evade immune responses. Once activated, plasminogen is converted to plasmin, a serine protease that can degrade fibrin and activate complement [39]. Plasmin has also been reported to increase the activity of many proteins including matrix metalloproteinases (MMP) and TGF-β which can alter host pathology and allow the tubercle bacillus to disseminate more readily [40]–[42]. Thrombospondin-4 (TSP4) prominently appears in both the paired and unpaired analysis. The thrombospondins are a family of extracellular matrix glycoproteins that mediate cell-to-cell and cell-to-matrix interactions. They have been reported to be involved in lung adhesion, fibrosis, neovascularization and cardiac tissue re-modeling [43]–[47], but to our knowledge they have not been associated with active TB. Fibroblast activation protein (SEPR) is also involved in collagen and extracellular matrix degradation. Additional MMPs and their endogenous inhibitors, tissue inhibitors of metalloproteinases (TIMPs) are both classes of enzymes involved in fibrosis and the proper formation of granulomatous inflammation [42], [48]–[53], tissue remodeling and turnover of extracellular matrix material in normal and pathological conditions [54]–[57]. The differential expression of these proteins may relate to underlying cavitary disease, resolution of liquefaction, local hypoxia and ultimate healing with fibrosis [57].

### Biomarkers of Angiogenesis and Coagulation

Angiogenesis is a complex biological phenomenon controlled by both positive and negative signals. The finding of 3 forms of vascular endothelial growth factor (VEGF) or its receptor among the top markers changing over time supports an intriguing role for angiogenesis and vascular remodeling and has recently been shown by others to be associated with TB [58]–[60]. We found significant changes in levels of proteins members (*e.g.* antithrombin III) of the coagulation cascades highlighting the importance of such cascades in the course of TB. There is a pro-coagulant state in TB [61]–[63] and others have shown that hematologic/coagulation factors can be biomarkers of TB infection [64]. Indeed careful study of lung histopathology of human and experimental MTB infection reveals areas of vasculitis [34] and microthrombi within vessels contributing to lesion formation [65].

### Biomarkers Associated with Disease Severity

When considering markers associated with the severity of disease a few proteins are worthy of specific comment. The levels of thrombospondin-2 (TSP-2), a protein regulating a variety of cell-matrix interactions [66] was found to be higher in those with more cavitary manifestations of disease. Three additional markers stood out in a logistic regression analysis of disease severity: DKK-1, serum amyloid P and adiponectin. DKK-1 an inhibitor of wnt signaling has also been shown to alter fibrosis [67] and expression is up-regulated in a Chlamydia infection model [68]. Serum amyloid P is a member (as is CRP) of the pentraxin family of proteins involved in pattern recognition and complement activation [59] and levels have been shown to correlate with disease severity and rapidity of burn wound healing [69]. Adiponectin is associated with metabolic syndrome and insulin resistance [70] and is known to be increased with decreased body fat so the finding of low levels with severe disease would argue against the association being attributed exclusively to low BMI in our patients. Adiponectin may be important in the lung, as receptors for this adipokine have been shown in the lung and low levels of adiponectin are associated with development of other serious pulmonary diseases (e.g. asthma) [71].

Many of the proteins identified throughout this work do not necessarily follow previous reports of mRNA transcript changes in TB [21] and suggest that each analytical method may be describing different cellular/host processes or reflect intrinsic differences between these two types of global analysis [72]. While drug treatment effects certainly could manifest with changes in serum proteins, we do not believe that all of such a large number of markers identified and the substantial magnitudes of the differential expression we observed are due to drug treatment alone but this remains to be studied. Proteins like cytochrome P450 enzymes (e.g. Cyp3A4) [73] and other drug metabolizing enzymes such as the superoxide dismutases (Sod1 and Sod2) and peroxiredoxins (Prdx1 and Prdx2) did not show any differential expression. In addition, we did not observe increases in typical markers of hepatotoxicity (aspartate aminotransferase) [74], [75]. Since rifampin is a broad spectrum antimicrobial agent we cannot conclusively rule out effects on gut microbial translocation, but it is useful to note that the samples used in this study were taken from a subset of the parent trial patients and were chosen to be free of co-morbidities like HIV infection, hepatitis C, diabetes, significant alcohol/drug use and cirrhosis; conditions which have been associated with increased microbial translocation. Even markers such as LBP and BPI have been associated with non-gram-negative infections and even with mycobacterial components [31], [32] lending support to our conclusions that the observed changes are unlikely to be the result of non-specific treatment effects on the microbiome but rather are due to treatment of TB disease itself. However, to answer this completely will require more rigorously designed clinical trials.

We recognize the limitations of this work. Small sample sizes, the omnipresence of pre-analytical variability, lack of suitable healthy-state and disease controls and studying a non-target organ (serum and not lung) are but a few of the challenges. Despite these limitations, the data contribute to further understanding of the complexity of changes accompanying TB disease.

We have presented overall false discovery rates for each list of proteins and listed individual q-values in each comparison. The latter are analogous to p-values in that they convey the significance of the result. For example, in a list of proteins q-values as large as 0.001 we expect on average of 1/1,000 proteins to eventually turn out to be “false discoveries”. Also, many of these markers share common pathways, regulons, or ontology, and their biological functions make sense in light of TB infection and disease (such as antimicrobial and tissue healing pathways). Finding such consistent themes within markers is intriguing and may further reduce the chance that these proteins are false discoveries.

Our current study has identified novel markers not previously reported to be associated with tuberculosis. Some of the biomarkers identified here are novel to the field of TB and will provide a new perspective on TB biology. With both familiar and new elements yielding intriguing findings, our study has contributed to the understanding of many aspects of TB infection as well as the complex adaptive systems involved in treatment of TB. We present this preliminary characterization of TB disease markers as an example of what can be done with such data, however, our findings still require future testing in properly designed validation studies using independent sample test sets with proper disease controls. Our study primarily assessed serum biomarkers of severity of disease and response to effective drug therapy, but we believe that our findings provide a platform for future investigation into the use of aptamer technology for diagnostic, drug efficacy and toxicity monitoring applications in TB.

## Methods

### Ethics Statement

TBTC Study 29 was approved by the institutional review boards of all participating institutions and the Center for Disease Control and Prevention (CDC), and registered with clinicaltrials.gov (Trial identifier: NCT00694629). Additionally, IRB approval was obtained from the Committee on Human Research of the University of California, San Francisco for the pilot evaluation of serum-based biomarkers for predicting response to TB treatment (H45279-34102-02A).

### Study Design and Data Analysis

TBTC Study 29 was a prospective, multicenter, open-label Phase 2B clinical trial comparing the antimicrobial activity and safety of standard TB therapy comprised of rifampin, isoniazid, pyrazinamide and ethambutol to that of an experimental regimen of rifapentine (10 mg/kg/dose), isoniazid, pyrazinamide and ethambutol in patients with drug-susceptible, smear-positive pulmonary TB. All patients signed a written informed consent. TBTC Study 29 methods and primary study results are published [4]. Briefly, all study participants were adults (age≥18 years) and had culture confirmed drug-susceptible TB diagnosed by sputum culture, agreed to HIV testing, had reasonably normal renal, hepatic and hematologic function, had no major co-morbidities. All received directly observed therapy (DOT) five of seven days a week, with up to 5 days of pre-enrollment treatment permitted before entry in the parent trial. As part of the biomarker sub-study of TBTC Study 29, serum was collected, processed and stored at baseline (time of enrollment into Study 29), and after 8 weeks (40 doses) of intensive phase therapy. For this project, additional end of treatment information including total duration of treatment and end of treatment cure status, was abstracted from patient charts by GM in Kampala, Uganda. For this pilot project, 39 HIV-uninfected study participants from the TBTC site in Kampala, Uganda without significant co-morbidities reported at enrollment were randomly selected by PN from participants enrolled from December, 2008 to July, 2009. Baseline and end of intensive phase treatment serum samples were analyzed. The SOMAscan assay was performed following standard protocols [1] and was carried out in full before receiving any clinical or demographic data. After all analyses were complete, data were submitted to the CDC after which clinical, radiographic and microbiologic data were shared with SomaLogic. After analysis of serum and receipt of the clinical data, a custom disease severity score was calculated based on chest radiograph (CXR) extent and cavitary disease (absent, diameter of all cavities summed less than or greater than 4 cm [5], sputum smear grade [6], days to detection of a positive culture in liquid media after inoculation and body mass index [weight in kg/(height in m)]^2^. A clinical spectrum of severity was determined to see if further refinement of disease severity could elucidate markers associated with more severe disease. Given the clinical importance of BMI [7] and bilateral cavitation [8] these factors were weighted by a factor of 2. Details regarding the calculation of the custom disease severity score (normalization, weighting, and calibration) can be found in Table S1, and the calculated custom disease severity scores are shown in Table S2 and Figure S1.

### SomaLogic Proteomic Methods

SomaLogic Inc., (Boulder, CO) performed all proteomic assessments and was blinded to the clinical characteristics of participating patients from whom sera was obtained for this study. Samples were analyzed in a single assay run and data validation was performed using internal assay controls without *a priori* knowledge of time point, clinical or microbiological details. SOMAmers were selected *in vitro* by the SELEX process (Systematic Evolution of Ligands by Exponential Enrichment) which consists of multiple rounds of selection, partitioning, and amplification [9], [10]. Success of SELEX and affinity of these binding reagents have greatly improved since using modified nucleotides to expand the chemistry of DNA for *in vitro* selection [11]. Version 2 SOMAscan assay, containing SOMAmers with sub-nanomolar K_d_’s, was used to test serum at three different concentrations (5%, 0.3%, 0.01%) to obtain accurate measurements within the dynamic range of the assay for low-, medium-, and high-abundant proteins, respectively. The integrity of serum samples provided for testing was monitored for known sample handling artifacts [3]. The SOMAmer-based proteomic assay consists of equilibrium binding of fluorophore-tagged SOMAmers and proteins in plasma or serum in solution and automated partitioning steps to capture only the SOMAmers that are in complexes with their cognate proteins [2]. The assay transforms the measurement of proteins into the measurement of the corresponding SOMAmers (DNA), via hybridization to an antisense probe array using a hybridization gasket slide with eight microarrays per slide (Agilent Technologies, Santa Clara, CA, USA). The liquid handling steps of the assay (protein binding) are performed by a Biomek robot, and the fluorescent signal generated in the hybridization step is captured. For this study, protein concentrations were reported in relative fluorescence units (RFU).

### Data Analysis and Statistical Method

Statistical analysis was performed using both Matlab™ and the R environment for statistical computing (http://www.r-project.org/). The Wilcoxon signed rank test was used for paired (within-subject) comparisons. To identify proteins differentially expressed between the baseline and week 8 time points. The Kolmogorov-Smirnoff (KS) test was used for unpaired comparisons between the baseline and week 8 populations. The KS statistic is an unsigned quantity, though to aid interpretation, we report a “signed” value using the sign to convey the directionality in the differential expression (that is, positive or negative KS distances for increased or decreased protein levels in a given comparison of interest). Multiple comparison corrections were performed using the false discovery rate (FDR) methodology [12] and we report both p-values and the associated FDR corrected “q-values” computed with the R package *q-value* [13]. We chose a nominal 0.01% false discovery rate to achieve a manageable number of proteins to consider while maintaining an acceptable expected number of false discoveries. The database for annotation, visualization and integrated discovery (DAVID) analysis was used for functional clustering and annotation (http://david.abcc.ncifcrf.gov/) [14]–[16].

## Supporting Information

Figure S1 **Disease severity custom score for 39 participants.** The custom score was calculated from eight parameters as shown in Table S1 and was based on the individual values shown in Table S2.
(TIF)

Figure S2 **DAVID analysis of paired sample data set.** Annotation Clusters, enrichment scores, and Bonferroni-corrected p-values are provided in spreadsheet format (XLSX).
(XLSX)

Figure S3 **Empirical cumulative distribution functions for the top 59 proteins from an unpaired analysis.** Proteins were measured at baseline (blue) and week 8 (red) in samples from *n* = 39 TB patients. The legend shows Bonferroni corrected p-value ranges associated with each color of the plot area.
(TIF)

Table S1 **Disease severity score calculation.** Eight parameters were combined into a custom disease severity score. CXRCLASS (Chest X-ray cavitation classification), 1–3, the higher the worse, absent (1), <4 cm (2), >4 cm (3); CXREXTNT (Chest X-ray extent of disease), A to C (converted to 1–3), limited (A), moderate (B), extensive (C); dtd_base (days to detection at baseline), the lower the worse (quicker detection if microbial burden is high); smearb (bacillus count in stained sputum sample via microscopy), 1–4, the higher the worse; bmi (body mass index at enrollment, kg/m^2^), the lower the worse (weight loss is a known effect of TB disease); anycav, 0 or 1 (1 = any cavitation reported at enrollment); bilatcav, 0 or 1 (1 = bilateral cavities reported at enrollment); bilatabn, 0 or 1 (1 = bilaternal abnormalities - adenopathy, pleural disease, infiltrates or cavities).
(DOCX)

Table S2 **Disease severity parameters and custom score calculation for 39 participants.** The parameters were normalized and weighed as shown in Table S1, and combined into a total score, then scaled to values from 0-1 to result in the custom score = (Total score +10)/16. A plot showing all participants and their custom score for disease severity is shown in Figure S1.
(DOCX)

Table S3 **Differential protein expression between baseline and week 8 in paired samples from** ***n*** **= 39 patients treated for pulmonary TB.** At a 0.01% false discovery rate (q <10^−4^) a total of 239 proteins were identified as differentially expressed using the Wilcoxon Sign Rank test. The intra-subject shifts are shown as the number of patients showing up- or down-regulation. Also shown are p-values for individual comparison and false discovery rate corrected q-values.
(DOCX)

Table S4 **Unpaired analysis of proteins showing differential expression between baseline and week 8 in** ***n*** **= 39 TB patients.** At a 0.1% false discovery rate (q <0.001), a total of 116 proteins were differentially expressed. The shift in expression is shown as a signed KS-distance, with a positive KS distance indicating up-regulation of the protein after 8 weeks of TB therapy.
(DOCX)
