# Supplementary figures and images for: Elucidating Novel Serum Biomarkers Associated with Pulmonary Tuberculosis Treatment
Source: PLoS One. 2013 Apr 18;8(4):e61002. doi: 10.1371/journal.pone.0061002 (PMC3630118; doi:10.1371/journal.pone.0061002)

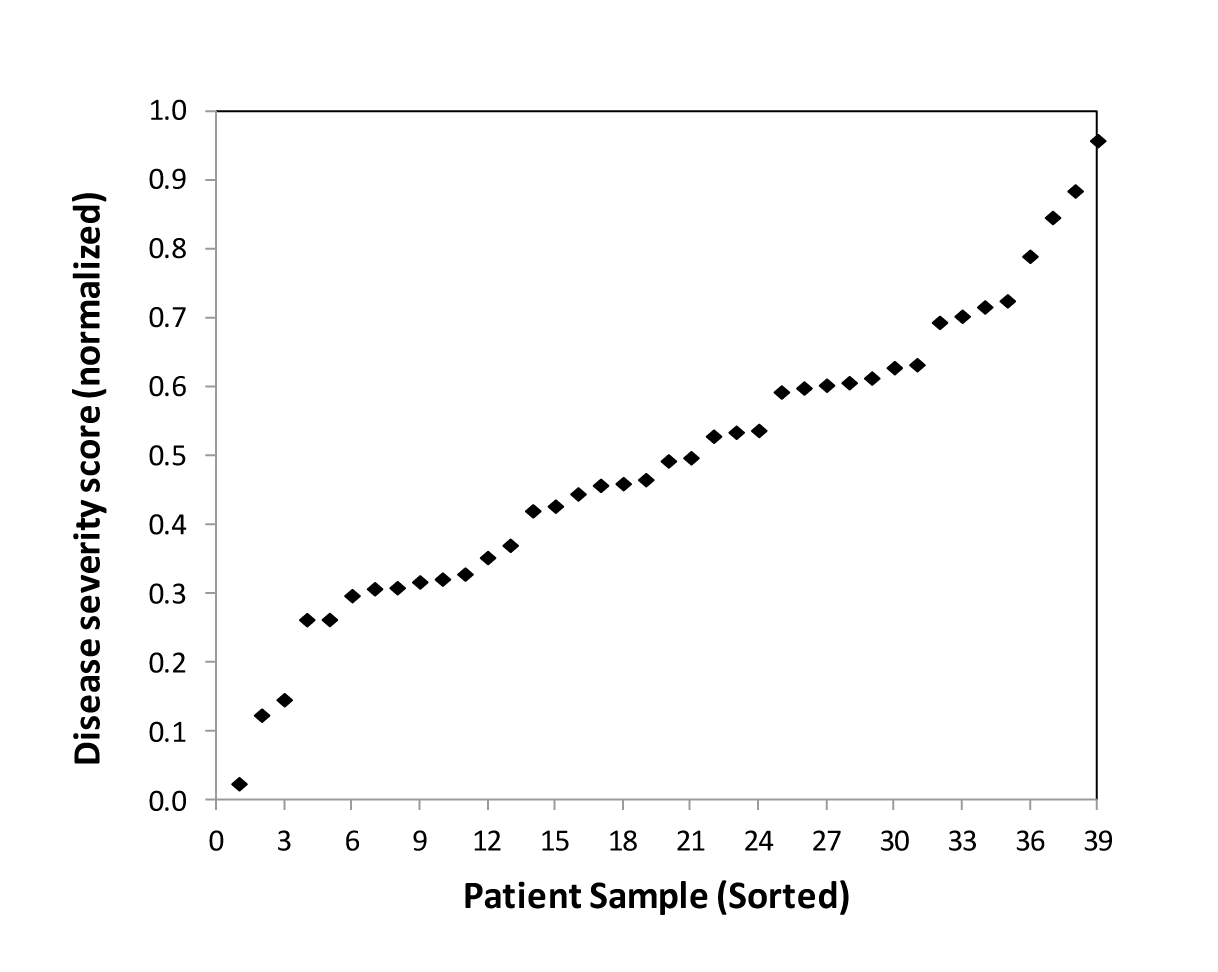

Supplement: Figure S1 — Disease severity custom score for 39 participants. The custom score was calculated from eight parameters as shown in Table S1 and was based on the individual values shown in Table S2. (TIF) [file pone.0061002.s001.tif]

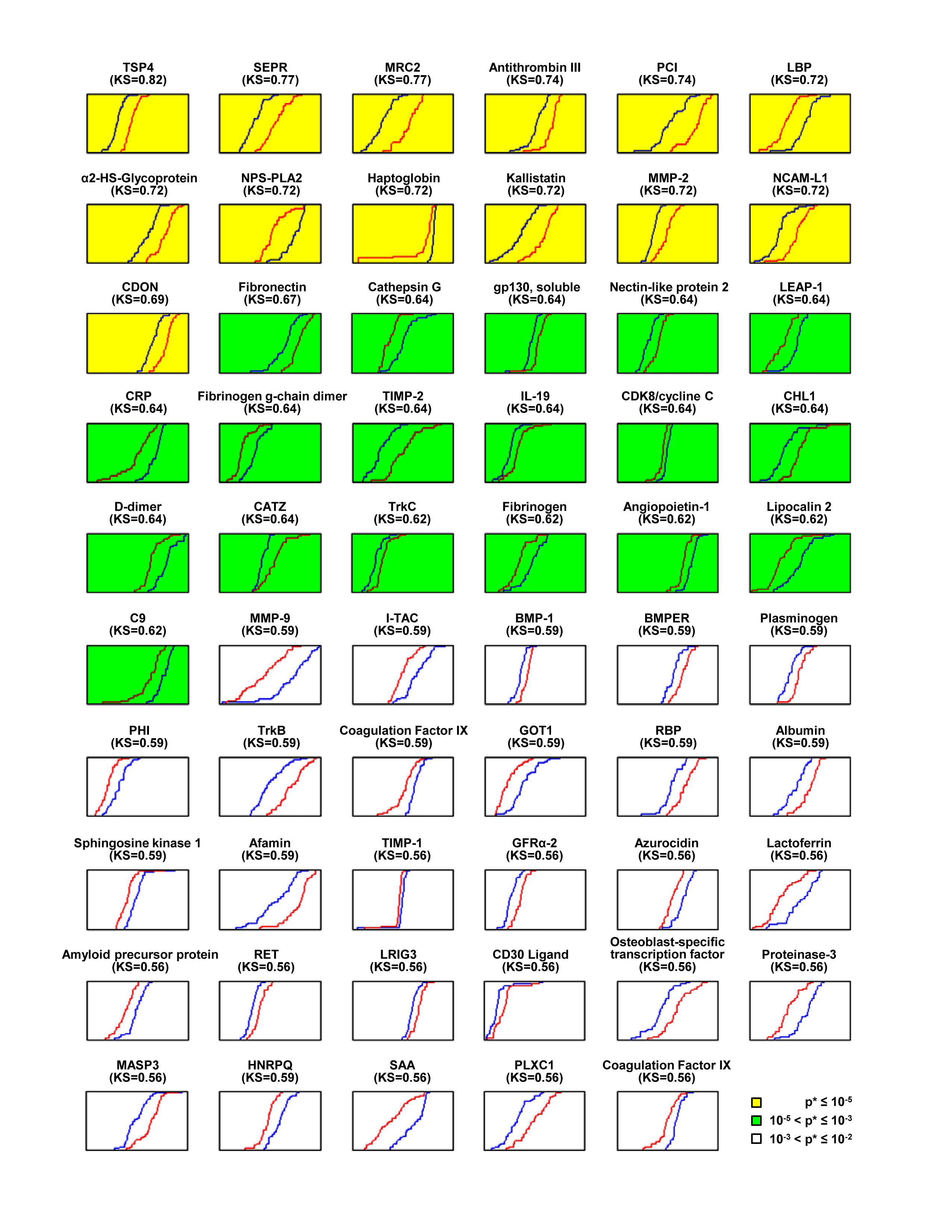

Supplement: Figure S3 — Empirical cumulative distribution functions for the top 59 proteins from an unpaired analysis. Proteins were measured at baseline (blue) and week 8 (red) in samples from n = 39 TB patients. The legend shows Bonferroni corrected p-value ranges associated with each color of the plot area. (TIF) [file pone.0061002.s003.tif]
