# Supplementary material for: Elucidating Novel Serum Biomarkers Associated with Pulmonary Tuberculosis Treatment
Source: PLoS One. 2013 Apr 18;8(4):e61002. doi: 10.1371/journal.pone.0061002 (PMC3630118; doi:10.1371/journal.pone.0061002)
Supplement: Table S1 — Disease severity score calculation. Eight parameters were combined into a custom disease severity score. CXRCLASS (Chest X-ray cavitation classification), 1–3, the higher the worse, absent (1), <4 cm (2), >4 cm (3); CXREXTNT (Chest X-ray extent of disease), A to C (converted to 1–3), limited (A), moderate (B), extensive (C); dtd_base (days to detection at baseline), the lower the worse (quicker detection if microbial burden is high); smearb (bacillus count in stained sputum sample via microscopy), 1–4, the higher the worse; bmi (body mass index at enrollment, kg/m2), the lower the worse (weight loss is a known effect of TB disease); anycav, 0 or 1 (1 = any cavitation reported at enrollment); bilatcav, 0 or 1 (1 = bilateral cavities reported at enrollment); bilatabn, 0 or 1 (1 = bilaternal abnormalities - adenopathy, pleural disease, infiltrates or cavities). (DOCX) [file pone.0061002.s004.docx]

| **Parameter** | **Range (min−max)** | **Normalization factor** | **Normalized Range (min−max)** | **Weigh factor** | **Scoring** |
| --- | --- | --- | --- | --- | --- |
| CXRCLASS | 1−3 | 1 | 1−3 | 1 | added |
| CXREXTNT | A-C (1−3) | 1 | 1−3 | 1 | added |
| dtd_base | 3.67−17.5 | 0.2 | 0.734 −3.5 | 1 | subtracted |
| smearb | 2−4 | 1 | 2−4 | 1 | added |
| bmi | 15.2−26.7 | 0.25 | 3.8−6.675 | 2 | subtracted |
| anycav | 0−1 | 1 | 0−1 | 1 | added |
| bilatcav | 0−1 | 1 | 0−1 | 2 | added |
| bilatabn | 0−1 | 1 | 0−1 | 1 | added |
