# Supplementary material for: Elucidating Novel Serum Biomarkers Associated with Pulmonary Tuberculosis Treatment
Source: PLoS One. 2013 Apr 18;8(4):e61002. doi: 10.1371/journal.pone.0061002 (PMC3630118; doi:10.1371/journal.pone.0061002)
Supplement: Table S2 — Disease severity parameters and custom score calculation for 39 participants. The parameters were normalized and weighed as shown in Table S1, and combined into a total score, then scaled to values from 0-1 to result in the custom score = (Total score +10)/16. A plot showing all participants and their custom score for disease severity is shown in Figure S1. (DOCX) [file pone.0061002.s005.docx]

| **PID** | **CXRCLASS** | **CXREXTNT** | **dtd_base** | **smearb** | **anycav** | **bilatcav** | **bilatabn** | **bmi** | **Custom Score** |
| --- | --- | --- | --- | --- | --- | --- | --- | --- | --- |
| 001 | 1 | 2 | 6.21 | 2 | 0 | 0 | 0 | 26.7 | 0.026 |
| 002 | 1 | 1 | 17.50 | 3 | 0 | 0 | 0 | 19 | 0.125 |
| 003 | 1 | 2 | 10.46 | 3 | 0 | 0 | 0 | 23.1 | 0.147 |
| 004 | 1 | 1 | 5.67 | 3 | 0 | 0 | 0 | 19.3 | 0.264 |
| 005 | 1 | 1 | 5.9 | 3 | 0 | 0 | 0 | 19.2 | 0.264 |
| 006 | 2 | 2 | 8.38 | 2 | 1 | 0 | 0 | 21.1 | 0.298 |
| 007 | 1 | 3 | 8.33 | 2 | 0 | 0 | 1 | 20.8 | 0.308 |
| 008 | 1 | 2 | 5.96 | 3 | 0 | 0 | 0 | 19.7 | 0.310 |
| 009 | 1 | 2 | 9.54 | 4 | 0 | 0 | 0 | 20 | 0.318 |
| 010 | 1 | 3 | 4.21 | 4 | 0 | 0 | 1 | 26 | 0.322 |
| 011 | 1 | 2 | 7.38 | 4 | 0 | 0 | 0 | 20.5 | 0.330 |
| 012 | 1 | 2 | 6.71 | 4 | 0 | 0 | 0 | 20 | 0.354 |
| 013 | 1 | 2 | 7.54 | 4 | 0 | 0 | 0 | 19.1 | 0.371 |
| 014 | 2 | 2 | 7.29 | 4 | 1 | 0 | 0 | 21.6 | 0.421 |
| 015 | 3 | 2 | 5.50 | 3 | 1 | 0 | 0 | 22.1 | 0.428 |
| 016 | 1 | 3 | 5.33 | 4 | 0 | 0 | 1 | 21.6 | 0.446 |
| 017 | 2 | 2 | 5.83 | 3 | 1 | 0 | 0 | 19 | 0.458 |
| 018 | 1 | 3 | 7.63 | 3 | 0 | 0 | 1 | 18.2 | 0.461 |
| 019 | 1 | 3 | 7.92 | 3 | 0 | 0 | 1 | 17.9 | 0.467 |
| 020 | 2 | 2 | 7.25 | 3 | 1 | 0 | 0 | 17.3 | 0.494 |
| 021 | 1 | 2 | 4.38 | 4 | 0 | 0 | 1 | 18.3 | 0.498 |
| 022 | 2 | 2 | 5.63 | 3 | 1 | 0 | 1 | 18.8 | 0.530 |
| 023 | 3 | 2 | 13.17 | 4 | 1 | 0 | 1 | 19.6 | 0.535 |
| 024 | 1 | 3 | 5.71 | 4 | 0 | 0 | 1 | 18.5 | 0.538 |
| 025 | 2 | 3 | 5.50 | 3 | 1 | 0 | 1 | 18.8 | 0.594 |
| 026 | 3 | 2 | 4.29 | 4 | 1 | 0 | 0 | 19.1 | 0.600 |
| 027 | 3 | 2 | 8.96 | 4 | 1 | 0 | 0 | 17.1 | 0.604 |
| 028 | 2 | 2 | 3.67 | 4 | 1 | 0 | 0 | 17.1 | 0.607 |
| 029 | 3 | 3 | 6.13 | 4 | 1 | 0 | 0 | 19.9 | 0.614 |
| 030 | 3 | 2 | 4.42 | 4 | 1 | 0 | 0 | 18.1 | 0.629 |
| 031 | 2 | 3 | 9.58 | 3 | 1 | 0 | 1 | 15.9 | 0.633 |
| 032 | 3 | 2 | 4.67 | 4 | 1 | 0 | 1 | 17.9 | 0.695 |
| 033 | 3 | 3 | 5.46 | 4 | 1 | 0 | 1 | 19.3 | 0.704 |
| 034 | 2 | 3 | 5.38 | 4 | 1 | 0 | 1 | 16.9 | 0.717 |
| 035 | 3 | 3 | 6.92 | 4 | 1 | 0 | 1 | 18 | 0.726 |
| 036 | 3 | 3 | 5.25 | 4 | 1 | 0 | 1 | 16.6 | 0.791 |
| 037 | 3 | 2 | 4.50 | 4 | 1 | 1 | 1 | 17.1 | 0.847 |
| 038 | 3 | 3 | 5.67 | 4 | 1 | 1 | 1 | 17.4 | 0.885 |
| 039 | 3 | 3 | 5.33 | 4 | 1 | 1 | 1 | 15.2 | 0.958 |
| min | 1 | 1 | 3.67 | 2 | 0 | 0 | 0 | 15.2 | 0.026 |
| max | 3 | 3 | 17.50 | 4 | 1 | 1 | 1 | 26.7 | 0.958 |
