# Supplementary material for: Elucidating Novel Serum Biomarkers Associated with Pulmonary Tuberculosis Treatment
Source: PLoS One. 2013 Apr 18;8(4):e61002. doi: 10.1371/journal.pone.0061002 (PMC3630118; doi:10.1371/journal.pone.0061002)
Supplement: Table S3 — Differential protein expression between baseline and week 8 in paired samples from n = 39 patients treated for pulmonary TB. At a 0.01% false discovery rate (q <10−4) a total of 239 proteins were identified as differentially expressed using the Wilcoxon Sign Rank test. The intra-subject shifts are shown as the number of patients showing up- or down-regulation. Also shown are p-values for individual comparison and false discovery rate corrected q-values. (DOCX) [file pone.0061002.s006.docx]

| **Rank** | **Target** | **Swiss Prot** | **Intrasubject Shift (baseline to week 8)** | | **p-value** | **q-value** |
| --- | --- | --- | --- | --- | --- | --- |
|  |  |  | **Up (*n*)** | **Down (*n*)** |  |  |
| 1) | TIMP-2 | P16035 | 39 |  | 5.3e-08 | 5.48e-07 |
| 2) | GFRα-2 | O00451 | 39 |  | 5.3e-08 | 5.48e-07 |
| 3) | MRC2 | Q9UBG0 | 39 |  | 5.3e-08 | 5.48e-07 |
| 4) | Haptoglobin, Mixed Type | P00738 |  | 39 | 5.3e-08 | 5.48e-07 |
| 5) | LBP | P18428 |  | 39 | 5.3e-08 | 5.48e-07 |
| 6) | Amyloid precursor protein | P05067 |  | 39 | 5.3e-08 | 5.48e-07 |
| 7) | BGH3 | Q15582 | 39 |  | 5.3e-08 | 5.48e-07 |
| 8) | TSP4 | P35443 | 39 |  | 5.3e-08 | 5.48e-07 |
| 9) | FETUB | Q9UGM5 | 39 |  | 5.3e-08 | 5.48e-07 |
| 10) | PCI | P05154 | 39 |  | 5.3e-08 | 5.48e-07 |
| 11) | Kallistatin | P29622 | 39 |  | 5.3e-08 | 5.48e-07 |
| 12) | α2-HS-Glycoprotein | P02765 | 39 |  | 5.3e-08 | 5.48e-07 |
| 13) | CHL1 | O00533 | 39 |  | 5.3e-08 | 5.48e-07 |
| 14) | CDON | Q4KMG0 | 39 |  | 5.3e-08 | 5.48e-07 |
| 15) | D-dimer | P02671, P02675, P02679 |  | 39 | 5.3e-08 | 5.48e-07 |
| 16) | MMP-1 | P03956 |  | 39 | 5.3e-08 | 5.48e-07 |
| 17) | contactin-1 | Q12860 | 38 |  | 5.7e-08 | 5.48e-07 |
| 18) | CD109 | Q6YHK3 | 38 |  | 5.7e-08 | 5.48e-07 |
| 19) | IGFBP-7 | Q16270 | 38 |  | 5.7e-08 | 5.48e-07 |
| 20) | Sphingosine kinase 1 | Q9NYA1 |  | 38 | 5.7e-08 | 5.48e-07 |
| 21) | CRP | P02741 |  | 38 | 5.7e-08 | 5.48e-07 |
| 22) | SEPR | Q12884 | 38 |  | 5.7e-08 | 5.48e-07 |
| 23) | TIMP-3 | P35625 |  | 38 | 6.1e-08 | 5.48e-07 |
| 24) | Lipocalin 2 | P80188 |  | 38 | 6.1e-08 | 5.48e-07 |
| 25) | NAP-2 | P02775 |  | 38 | 6.1e-08 | 5.48e-07 |
| 26) | Nectin-like protein 2 | Q9BY67 | 38 |  | 6.1e-08 | 5.48e-07 |
| 27) | Proteinase-3 | P24158 |  | 38 | 6.1e-08 | 5.48e-07 |
| 28) | PDGF-BB | P01127 |  | 38 | 6.1e-08 | 5.48e-07 |
| 29) | MMP-2 | P08253 | 38 |  | 6.1e-08 | 5.48e-07 |
| 30) | TIMP-1 | P01033 |  | 37 | 6.6e-08 | 5.48e-07 |
| 31) | ROR1 | Q01973 | 37 |  | 6.6e-08 | 5.48e-07 |
| 32) | IGFBP-6 | P24592 | 37 |  | 6.6e-08 | 5.48e-07 |
| 33) | PAI-1 | P05121 |  | 38 | 6.6e-08 | 5.48e-07 |
| 34) | Protein C | P04070 | 37 |  | 6.6e-08 | 5.48e-07 |
| 35) | C9 | P02748 |  | 38 | 6.6e-08 | 5.48e-07 |
| 36) | GDF-9 | O60383 |  | 37 | 6.6e-08 | 5.48e-07 |
| 37) | Carbonic anhydrase 6 | P23280 | 37 |  | 6.6e-08 | 5.48e-07 |
| 38) | RBP | P02753 | 37 |  | 6.6e-08 | 5.48e-07 |
| 39) | Albumin | P02768 | 38 |  | 6.6e-08 | 5.48e-07 |
| 40) | Fibronectin | P02751 | 37 |  | 6.6e-08 | 5.48e-07 |
| 41) | Antithrombin III | P01008 | 38 |  | 7.2e-08 | 5.48e-07 |
| 42) | a1-Antitrypsin | P01009 |  | 37 | 7.2e-08 | 5.48e-07 |
| 43) | HRG | P04196 | 38 |  | 7.2e-08 | 5.48e-07 |
| 44) | Angiopoietin-1 | Q15389 |  | 38 | 7.8e-08 | 5.48e-07 |
| 45) | ATS13 | Q76LX8 | 37 |  | 7.8e-08 | 5.48e-07 |
| 46) | Coagulation Factor VII | P08709 | 37 |  | 7.8e-08 | 5.48e-07 |
| 47) | Afamin | P43652 | 38 |  | 7.8e-08 | 5.48e-07 |
| 48) | TrkB | Q16620 | 37 |  | 7.8e-08 | 5.48e-07 |
| 49) | GOT1 | P17174 |  | 38 | 7.8e-08 | 5.48e-07 |
| 50) | Azurocidin | P20160 |  | 38 | 8.4e-08 | 5.80e-07 |
| 51) | NCAM-L1 | P32004 | 38 |  | 9.1e-08 | 6.03e-07 |
| 52) | PLXC1 | O60486 | 36 |  | 9.1e-08 | 6.03e-07 |
| 53) | I-TAC | O14625 |  | 38 | 1.1e-07 | 6.41e-07 |
| 54) | CYTF | O76096 |  | 37 | 1.1e-07 | 6.41e-07 |
| 55) | BPI | P17213 |  | 36 | 1.1e-07 | 6.41e-07 |
| 56) | HNRPQ | O60506 |  | 36 | 1.1e-07 | 6.41e-07 |
| 57) | PHI | P06744 |  | 36 | 1.1e-07 | 6.41e-07 |
| 58) | Cathepsin G | P08311 |  | 36 | 1.1e-07 | 6.47e-07 |
| 59) | Osteoblast-specific transcription factor 2 | Q13950 | 37 |  | 1.1e-07 | 6.47e-07 |
| 60) | SAA | P02735 |  | 38 | 1.1e-07 | 6.47e-07 |
| 61) | TXD12 | O95881 |  | 36 | 1.1e-07 | 6.47e-07 |
| 62) | gp130, soluble | P40189 | 36 |  | 1.2e-07 | 6.87e-07 |
| 63) | ITI heavy chain H4 | Q14624 |  | 36 | 1.3e-07 | 7.30e-07 |
| 64) | CDK8/cyclin C | P49336, P24863 |  | 36 | 1.4e-07 | 7.63e-07 |
| 65) | VEGF121 | P15692 |  | 38 | 1.4e-07 | 7.63e-07 |
| 66) | LRIG3 | Q6UXM1 | 36 |  | 1.5e-07 | 7.98e-07 |
| 67) | MAPK14 | Q16539 |  | 36 | 1.5e-07 | 7.98e-07 |
| 68) | PGRP-S | O75594 |  | 36 | 1.7e-07 | 8.24e-07 |
| 69) | RGM-C | Q6ZVN8 | 36 |  | 1.7e-07 | 8.24e-07 |
| 70) | Fibrinogen g-chain dimer | P02679 |  | 37 | 1.7e-07 | 8.24e-07 |
| 71) | MMP-9 | P14780 |  | 36 | 1.8e-07 | 8.52e-07 |
| 72) | Thyroxine-Binding Globulin | P05543 | 35 |  | 1.8e-07 | 8.52e-07 |
| 73) | Cadherin-5 | P33151 | 35 |  | 1.8e-07 | 8.52e-07 |
| 74) | NPS-PLA2 | P14555 |  | 37 | 1.9e-07 | 9.07e-07 |
| 75) | NAP-2 | P02775 |  | 36 | 2.1e-07 | 9.51e-07 |
| 76) | FN1.3 | P02751 | 34 |  | 2.1e-07 | 9.51e-07 |
| 77) | Protease nexin I | P07093 |  | 38 | 2.3e-07 | 9.99e-07 |
| 78) | Plasminogen | P00747 | 34 |  | 2.3e-07 | 9.99e-07 |
| 79) | Lactoferrin | P02788 |  | 36 | 2.4e-07 | 1.04e-06 |
| 80) | Dkk-4 | Q9UBT3 |  | 35 | 2.4e-07 | 1.04e-06 |
| 81) | PDGF-AA | P04085 |  | 36 | 2.4e-07 | 1.04e-06 |
| 82) | Gelsolin | P06396 | 33 |  | 2.6e-07 | 1.10e-06 |
| 83) | Macrophage mannose receptor | P22897 |  | 35 | 2.8e-07 | 1.15e-06 |
| 84) | Alkaline phosphatase, bone | P05186 |  | 35 | 2.8e-07 | 1.15e-06 |
| 85) | FUT5 | Q11128 |  | 34 | 2.8e-07 | 1.15e-06 |
| 86) | Apo A-I | P02647 | 35 |  | 3.0e-07 | 1.22e-06 |
| 87) | SDF-1a | P48061 | 35 |  | 3.3e-07 | 1.27e-06 |
| 88) | MIA | Q16674 | 34 |  | 3.3e-07 | 1.27e-06 |
| 89) | GDF-11 | O95390 | 37 |  | 3.3e-07 | 1.27e-06 |
| 90) | VEGF | P15692 |  | 37 | 3.5e-07 | 1.31e-06 |
| 91) | MPIF-1 | P55773 |  | 37 | 3.5e-07 | 1.31e-06 |
| 92) | FN1.4 | P02751 | 34 |  | 3.5e-07 | 1.31e-06 |
| 93) | Cofilin-1 | P23528 |  | 36 | 3.5e-07 | 1.31e-06 |
| 94) | Contactin-4 | Q8IWV2 | 37 |  | 3.8e-07 | 1.39e-06 |
| 95) | Calpain I | P07384, P04632 |  | 37 | 4.1e-07 | 1.47e-06 |
| 96) | RET | P07949 | 34 |  | 4.1e-07 | 1.47e-06 |
| 97) | Contactin-5 | O94779 | 35 |  | 4.4e-07 | 1.53e-06 |
| 98) | LEAP-1 | P81172 |  | 36 | 4.4e-07 | 1.53e-06 |
| 99) | MASP3 | P48740 | 33 |  | 4.4e-07 | 1.53e-06 |
| 100) | LSAMP | Q13449 | 35 |  | 4.7e-07 | 1.60e-06 |
| 101) | BMPER | Q8N8U9 | 35 |  | 4.7e-07 | 1.60e-06 |
| 102) | PAFAH | Q13093 | 36 |  | 4.7e-07 | 1.60e-06 |
| 103) | C2 | P06681 |  | 35 | 5.1e-07 | 1.67e-06 |
| 104) | HGFA | Q04756 | 38 |  | 5.1e-07 | 1.67e-06 |
| 105) | CTAP-III | P02775 |  | 35 | 5.1e-07 | 1.67e-06 |
| 106) | Protein S | P07225 |  | 34 | 5.4e-07 | 1.78e-06 |
| 107) | IGFBP-3 | P17936 | 33 |  | 5.9e-07 | 1.84e-06 |
| 108) | HSP 90a | P07900 |  | 34 | 5.9e-07 | 1.84e-06 |
| 109) | TrkC | Q16288 | 36 |  | 5.9e-07 | 1.84e-06 |
| 110) | PSA-ACT | P07288, P01011 |  | 36 | 5.9e-07 | 1.84e-06 |
| 111) | a2-Macroglobulin | P01023 | 33 |  | 6.3e-07 | 1.95e-06 |
| 112) | IP-10 | P02778 |  | 36 | 6.3e-07 | 1.95e-06 |
| 113) | Resistin | Q9HD89 |  | 33 | 6.8e-07 | 2.04e-06 |
| 114) | RASA1 | P20936 |  | 37 | 6.8e-07 | 2.04e-06 |
| 115) | CATZ | Q9UBR2 | 34 |  | 6.8e-07 | 2.04e-06 |
| 116) | ZAP70 | P43403 |  | 35 | 7.8e-07 | 2.31e-06 |
| 117) | Factor B | P00751 |  | 36 | 7.8e-07 | 2.31e-06 |
| 118) | TGF-b R III | Q03167 | 35 |  | 8.4e-07 | 2.44e-06 |
| 119) | CAPG | P40121 |  | 35 | 8.4e-07 | 2.44e-06 |
| 120) | Flt3 ligand | P49771 |  | 33 | 9.0e-07 | 2.60e-06 |
| 121) | ERBB1 | P00533 | 35 |  | 9.7e-07 | 2.74e-06 |
| 122) | Gro-b | P19875 |  | 35 | 9.7e-07 | 2.74e-06 |
| 123) | bFGF-R | P11362 | 35 |  | 1.0e-06 | 2.92e-06 |
| 124) | BMP-1 | P13497 | 36 |  | 1.1e-06 | 3.09e-06 |
| 125) | Angiostatin | P00747 | 33 |  | 1.1e-06 | 3.09e-06 |
| 126) | SAP | P02743 |  | 34 | 1.3e-06 | 3.50e-06 |
| 127) | Coagulation Factor IX | P00740 |  | 36 | 1.3e-06 | 3.50e-06 |
| 128) | IGFBP-5 | P24593 | 36 |  | 1.4e-06 | 3.66e-06 |
| 129) | CNDP1 | Q96KN2 | 36 |  | 1.4e-06 | 3.66e-06 |
| 130) | Cadherin-2 | P19022 |  | 35 | 1.4e-06 | 3.66e-06 |
| 131) | VEGF sR3 | P35916 |  | 34 | 1.5e-06 | 3.87e-06 |
| 132) | Siglec-9 | Q9Y336 |  | 33 | 1.5e-06 | 3.87e-06 |
| 133) | IDUA | P35475 | 35 |  | 1.7e-06 | 4.42e-06 |
| 134) | suPAR | Q03405 |  | 33 | 1.8e-06 | 4.66e-06 |
| 135) | Coagulation Factor IX | P00740 |  | 36 | 1.8e-06 | 4.66e-06 |
| 136) | CD30 Ligand | P32971 | 32 |  | 1.9e-06 | 4.92e-06 |
| 137) | 14-3-3 eta | Q04917 |  | 33 | 1.9e-06 | 4.92e-06 |
| 138) | TIG2 | Q99969 |  | 34 | 2.2e-06 | 5.61e-06 |
| 139) | HGF | P14210 |  | 36 | 2.4e-06 | 5.92e-06 |
| 140) | TNF sR-II | P20333 |  | 33 | 2.4e-06 | 5.92e-06 |
| 141) | Factor I | P05156 |  | 37 | 2.6e-06 | 6.13e-06 |
| 142) | OLR1 | P78380 |  | 33 | 2.6e-06 | 6.13e-06 |
| 143) | Thrombin | P00734 |  | 31 | 2.6e-06 | 6.13e-06 |
| 144) | Collectin Kidney 1 | Q9BWP8 | 35 |  | 2.6e-06 | 6.13e-06 |
| 145) | Endoglin | P17813 | 35 |  | 2.6e-06 | 6.13e-06 |
| 146) | ON | P09486 |  | 32 | 2.7e-06 | 6.43e-06 |
| 147) | CATC | P53634 |  | 32 | 2.7e-06 | 6.43e-06 |
| 148) | DKK3 | Q9UBP4 | 32 |  | 2.7e-06 | 6.43e-06 |
| 149) | Fibrinogen | P02671, P02675, P02679 |  | 34 | 2.9e-06 | 6.83e-06 |
| 150) | MMP-8 | P22894 |  | 34 | 3.1e-06 | 7.17e-06 |
| 151) | TPSB2 | P20231 | 33 |  | 3.1e-06 | 7.17e-06 |
| 152) | CK-MB | P12277, P06732 | 33 |  | 3.1e-06 | 7.17e-06 |
| 153) | BASI | P35613 | 33 |  | 3.4e-06 | 7.62e-06 |
| 154) | Adiponectin | Q15848 | 33 |  | 3.6e-06 | 8.10e-06 |
| 155) | Myeloperoxidase | P05164 |  | 34 | 3.9e-06 | 8.44e-06 |
| 156) | Aurora kinase A | O14965 |  | 34 | 3.9e-06 | 8.44e-06 |
| 157) | IL-18 Rα | Q13478 |  | 32 | 3.9e-06 | 8.44e-06 |
| 158) | 14-3-3 protein gamma | P61981 |  | 33 | 3.9e-06 | 8.44e-06 |
| 159) | Ck-b-8-1 | P55773 |  | 35 | 4.1e-06 | 8.97e-06 |
| 160) | FCG3B | O75015 |  | 33 | 4.4e-06 | 9.54e-06 |
| 161) | MIP-1a | P10147 |  | 32 | 4.7e-06 | 9.95e-06 |
| 162) | TFPI | P10646 |  | 32 | 4.7e-06 | 9.95e-06 |
| 163) | NCAM-120 | P13591 | 33 |  | 4.7e-06 | 9.95e-06 |
| 164) | TSG-6 | P98066 |  | 33 | 4.7e-06 | 9.95e-06 |
| 165) | HAI-1 | O43278 | 34 |  | 5.0e-06 | 1.06e-05 |
| 166) | CNTFR alpha | P26992 | 31 |  | 5.4e-06 | 1.10e-05 |
| 167) | Factor D | P00746 | 35 |  | 5.4e-06 | 1.10e-05 |
| 168) | IL-17 RC | Q8NAC3 |  | 35 | 5.4e-06 | 1.10e-05 |
| 169) | BOC | Q9BWV1 | 34 |  | 5.4e-06 | 1.10e-05 |
| 170) | Spondin-1 | Q9HCB6 | 35 |  | 5.8e-06 | 1.17e-05 |
| 171) | RGMB | Q6NW40 | 31 |  | 6.1e-06 | 1.23e-05 |
| 172) | Lysozyme | P61626 |  | 33 | 6.1e-06 | 1.23e-05 |
| 173) | C1QBP | Q07021 |  | 31 | 6.1e-06 | 1.23e-05 |
| 174) | Gro-g | P19876 |  | 33 | 7.0e-06 | 1.39e-05 |
| 175) | CD5L | O43866 |  | 31 | 8.0e-06 | 1.55e-05 |
| 176) | MAPK2 | P49137 |  | 31 | 8.0e-06 | 1.55e-05 |
| 177) | C6 | P13671 |  | 33 | 8.0e-06 | 1.55e-05 |
| 178) | JAK2 | O60674 |  | 33 | 8.0e-06 | 1.55e-05 |
| 179) | Apo E3 | P02649 | 34 |  | 8.5e-06 | 1.65e-05 |
| 180) | Apo B | P04114 | 32 |  | 9.1e-06 | 1.73e-05 |
| 181) | LYVE1 | Q9Y5Y7 | 32 |  | 9.1e-06 | 1.73e-05 |
| 182) | Endocan | Q9NQ30 |  | 34 | 9.1e-06 | 1.73e-05 |
| 183) | ASAHL | Q02083 | 34 |  | 9.7e-06 | 1.84e-05 |
| 184) | PBEF | P43490 |  | 33 | 1.0e-05 | 1.95e-05 |
| 185) | CD23 | P06734 | 35 |  | 1.1e-05 | 2.07e-05 |
| 186) | WFKN2 | Q8TEU8 | 32 |  | 1.2e-05 | 2.17e-05 |
| 187) | RGMA | Q96B86 |  | 32 | 1.2e-05 | 2.17e-05 |
| 188) | ENTP3 | O75355 |  | 35 | 1.2e-05 | 2.17e-05 |
| 189) | PF-4 | P02776 |  | 31 | 1.3e-05 | 2.29e-05 |
| 190) | HPG- | P15428 |  | 31 | 1.3e-05 | 2.29e-05 |
| 191) | ZAP70 | P43403 |  | 34 | 1.3e-05 | 2.42e-05 |
| 192) | Cystatin C | P01034 | 31 |  | 1.4e-05 | 2.56e-05 |
| 193) | C3d | P01024 |  | 33 | 1.4e-05 | 2.56e-05 |
| 194) | AIF1 | P55008 |  | 34 | 1.5e-05 | 2.67e-05 |
| 195) | URB | Q76M96 | 33 |  | 1.5e-05 | 2.67e-05 |
| 196) | GSK-3 beta | P49841 |  | 32 | 1.5e-05 | 2.67e-05 |
| 197) | MATN2 | O00339 | 33 |  | 1.5e-05 | 2.67e-05 |
| 198) | Ephrin-A5 | P52803 | 32 |  | 1.6e-05 | 2.83e-05 |
| 199) | CYTD | P28325 | 35 |  | 1.8e-05 | 3.16e-05 |
| 200) | HDGR2 | Q7Z4V5 |  | 32 | 1.8e-05 | 3.16e-05 |
| 201) | PH | P01298 |  | 35 | 1.8e-05 | 3.16e-05 |
| 202) | TNF sR-I | P19438 |  | 30 | 2.0e-05 | 3.28e-05 |
| 203) | IL-2 sRa | P01589 |  | 34 | 2.0e-05 | 3.28e-05 |
| 204) | IFN-g R1 | P15260 | 33 |  | 2.0e-05 | 3.28e-05 |
| 205) | DHH | O43323 |  | 32 | 2.0e-05 | 3.28e-05 |
| 206) | Coactosin-like protein | Q14019 |  | 32 | 2.0e-05 | 3.28e-05 |
| 207) | SCF sR | P10721 | 34 |  | 2.1e-05 | 3.46e-05 |
| 208) | HSP 70 | P08107 |  | 34 | 2.1e-05 | 3.46e-05 |
| 209) | IL-13 | P35225 | 34 |  | 2.2e-05 | 3.65e-05 |
| 210) | DKK1 | O94907 |  | 34 | 2.2e-05 | 3.65e-05 |
| 211) | M-CSF R | P07333 | 31 |  | 2.4e-05 | 3.84e-05 |
| 212) | Bcl-2 | P10415 |  | 31 | 2.4e-05 | 3.84e-05 |
| 213) | TARC | Q92583 |  | 32 | 2.5e-05 | 4.07e-05 |
| 214) | Apo E | P02649 | 33 |  | 2.7e-05 | 4.23e-05 |
| 215) | CD36 ANTIGEN | P16671 | 33 |  | 2.7e-05 | 4.23e-05 |
| 216) | IL-19 | Q9UHD0 | 34 |  | 2.7e-05 | 4.23e-05 |
| 217) | Kininogen, HMW, Single Chain | P01042 | 32 |  | 2.7e-05 | 4.23e-05 |
| 218) | IL-1a | P01583 |  | 33 | 2.7e-05 | 4.23e-05 |
| 219) | Apo E4 | P02649 | 33 |  | 2.8e-05 | 4.48e-05 |
| 220) | SDF-1b | P48061 | 32 |  | 3.4e-05 | 5.33e-05 |
| 221) | Macrophage scavenger receptor | P21757 |  | 33 | 3.4e-05 | 5.33e-05 |
| 222) | Midkine | P21741 |  | 31 | 3.6e-05 | 5.59e-05 |
| 223) | IGF-I | P05019 | 30 |  | 3.6e-05 | 5.59e-05 |
| 224) | AK1A1 | P14550 |  | 33 | 3.6e-05 | 5.59e-05 |
| 225) | EDAR | Q9UNE0 | 31 |  | 4.1e-05 | 6.25e-05 |
| 226) | Hemoglobin | P69905, P68871 | 33 |  | 4.1e-05 | 6.25e-05 |
| 227) | GP1BA | P07359 |  | 32 | 4.3e-05 | 6.61e-05 |
| 228) | BARK1 | P25098 |  | 31 | 4.6e-05 | 6.99e-05 |
| 229) | Calpastatin | P20810 | 30 |  | 5.2e-05 | 7.81e-05 |
| 230) | OBCAM | Q14982 |  | 30 | 5.2e-05 | 7.81e-05 |
| 231) | Growth hormone receptor | P10912 | 32 |  | 5.5e-05 | 8.25e-05 |
| 232) | pTEN | P60484 |  | 33 | 5.8e-05 | 8.68e-05 |
| 233) | IF4G2 | P78344 |  | 31 | 5.8e-05 | 8.68e-05 |
| 234) | GASP-2 | Q96D09 | 31 |  | 6.2e-05 | 9.06e-05 |
| 235) | Cystatin M | Q15828 | 29 |  | 6.2e-05 | 9.06e-05 |
| 236) | dopa decarboxylase | P20711 | 34 |  | 6.2e-05 | 9.06e-05 |
| 237) | LCMT1 | Q9UIC8 |  | 30 | 6.2e-05 | 9.06e-05 |
| 238) | Myoglobin | P02144 |  | 30 | 6.6e-05 | 9.53e-05 |
| 239) | NRP1 | O14786 |  | 32 | 6.6e-05 | 9.53e-05 |
