# Supplementary material for: Elucidating Novel Serum Biomarkers Associated with Pulmonary Tuberculosis Treatment
Source: PLoS One. 2013 Apr 18;8(4):e61002. doi: 10.1371/journal.pone.0061002 (PMC3630118; doi:10.1371/journal.pone.0061002)
Supplement: Table S4 — Unpaired analysis of proteins showing differential expression between baseline and week 8 in n = 39 TB patients. At a 0.1% false discovery rate (q <0.001), a total of 116 proteins were differentially expressed. The shift in expression is shown as a signed KS-distance, with a positive KS distance indicating up-regulation of the protein after 8 weeks of TB therapy. (DOCX) [file pone.0061002.s007.docx]

|  | **Target** | **Swiss Prot** | **KS-distance (Signed)** | **p-value** | **q-value** |
| --- | --- | --- | --- | --- | --- |
| 1) | TSP4 | P35443 | 0.821 | 1.37e-12 | 1.06e-09 |
| 2) | SEPR | Q12884 | 0.769 | 4.08e-11 | 1.05e-08 |
| 3) | MRC2 | Q9UBG0 | 0.769 | 4.08e-11 | 1.05e-08 |
| 4) | Antithrombin III | P01008 | 0.744 | 2.05e-10 | 3.15e-08 |
| 5) | PCI | P05154 | 0.744 | 2.05e-10 | 3.15e-08 |
| 6) | LBP | P18428 | -0.718 | 9.73e-10 | 6.25e-08 |
| 7) | a2-HS-Glycoprotein | P02765 | 0.718 | 9.73e-10 | 6.25e-08 |
| 8) | NPS-PLA2 | P14555 | -0.718 | 9.73e-10 | 6.25e-08 |
| 9) | Haptoglobin, Mixed Type | P00738 | -0.718 | 9.73e-10 | 6.25e-08 |
| 10) | Kallistatin | P29622 | 0.718 | 9.73e-10 | 6.25e-08 |
| 11) | MMP-2 | P08253 | 0.718 | 9.73e-10 | 6.25e-08 |
| 12) | NCAM-L1 | P32004 | 0.718 | 9.73e-10 | 6.25e-08 |
| 13) | CDON | Q4KMG0 | 0.692 | 4.38e-09 | 2.60e-07 |
| 14) | Fibronectin | P02751 | 0.667 | 1.87e-08 | 1.03e-06 |
| 15) | Cathepsin G | P08311 | -0.641 | 7.53e-08 | 2.23e-06 |
| 16) | gp130, soluble | P40189 | 0.641 | 7.53e-08 | 2.23e-06 |
| 17) | Nectin-like protein 2 | Q9BY67 | 0.641 | 7.53e-08 | 2.23e-06 |
| 18) | LEAP-1 | P81172 | -0.641 | 7.53e-08 | 2.23e-06 |
| 19) | CRP | P02741 | -0.641 | 7.53e-08 | 2.23e-06 |
| 20) | Fibrinogen g-chain dimer | P02679 | -0.641 | 7.53e-08 | 2.23e-06 |
| 21) | TIMP-2 | P16035 | 0.641 | 7.53e-08 | 2.23e-06 |
| 22) | IL-19 | Q9UHD0 | 0.641 | 7.53e-08 | 2.23e-06 |
| 23) | CDK8/cyclin C | P49336, P24863 | -0.641 | 7.53e-08 | 2.23e-06 |
| 24) | CHL1 | O00533 | 0.641 | 7.53e-08 | 2.23e-06 |
| 25) | D-dimer | P02671, P02675, P02679 | -0.641 | 7.53e-08 | 2.23e-06 |
| 26) | CATZ | Q9UBR2 | 0.641 | 7.53e-08 | 2.23e-06 |
| 27) | TrkC | Q16288 | 0.615 | 2.88e-07 | 7.15e-06 |
| 28) | Fibrinogen | P02671, P02675, P02679 | -0.615 | 2.88e-07 | 7.15e-06 |
| 29) | Angiopoietin-1 | Q15389 | -0.615 | 2.88e-07 | 7.15e-06 |
| 30) | Lipocalin 2 | P80188 | -0.615 | 2.88e-07 | 7.15e-06 |
| 31) | C9 | P02748 | -0.615 | 2.88e-07 | 7.15e-06 |
| 32) | MMP-9 | P14780 | -0.590 | 1.04e-06 | 1.82e-05 |
| 33) | I-TAC | O14625 | -0.590 | 1.04e-06 | 1.82e-05 |
| 34) | BMP-1 | P13497 | 0.590 | 1.04e-06 | 1.82e-05 |
| 35) | BMPER | Q8N8U9 | 0.590 | 1.04e-06 | 1.82e-05 |
| 36) | Plasminogen | P00747 | 0.590 | 1.04e-06 | 1.82e-05 |
| 37) | PHI | P06744 | -0.590 | 1.04e-06 | 1.82e-05 |
| 38) | TrkB | Q16620 | 0.590 | 1.04e-06 | 1.82e-05 |
| 39) | Coagulation Factor IX | P00740 | -0.590 | 1.04e-06 | 1.82e-05 |
| 40) | GOT1 | P17174 | -0.590 | 1.04e-06 | 1.82e-05 |
| 41) | RBP | P02753 | 0.590 | 1.04e-06 | 1.82e-05 |
| 42) | Albumin | P02768 | 0.590 | 1.04e-06 | 1.82e-05 |
| 43) | Sphingosine kinase 1 | Q9NYA1 | -0.590 | 1.04e-06 | 1.82e-05 |
| 44) | Afamin | P43652 | 0.590 | 1.04e-06 | 1.82e-05 |
| 45) | TIMP-1 | P01033 | -0.564 | 3.56e-06 | 4.58e-05 |
| 46) | GFRa-2 | O00451 | 0.564 | 3.56e-06 | 4.58e-05 |
| 47) | Azurocidin | P20160 | -0.564 | 3.56e-06 | 4.58e-05 |
| 48) | Lactoferrin | P02788 | -0.564 | 3.56e-06 | 4.58e-05 |
| 49) | amyloid precursor protein | P05067 | -0.564 | 3.56e-06 | 4.58e-05 |
| 50) | RET | P07949 | 0.564 | 3.56e-06 | 4.58e-05 |
| 51) | LRIG3 | Q6UXM1 | 0.564 | 3.56e-06 | 4.58e-05 |
| 52) | CD30 Ligand | P32971 | 0.564 | 3.56e-06 | 4.58e-05 |
| 53) | Osteoblast-specific transcription factor 2 | Q13950 | 0.564 | 3.56e-06 | 4.58e-05 |
| 54) | Proteinase-3 | P24158 | -0.564 | 3.56e-06 | 4.58e-05 |
| 55) | MASP3 | P48740 | 0.564 | 3.56e-06 | 4.58e-05 |
| 56) | HNRPQ | O60506 | -0.564 | 3.56e-06 | 4.58e-05 |
| 57) | SAA | P02735 | -0.564 | 3.56e-06 | 4.58e-05 |
| 58) | PLXC1 | O60486 | 0.564 | 3.56e-06 | 4.58e-05 |
| 59) | Coagulation Factor IX | P00740 | -0.564 | 3.56e-06 | 4.58e-05 |
| 60) | CAPG | P40121 | -0.564 | 3.56e-06 | 4.58e-05 |
| 61) | HSP 90a | P07900 | -0.538 | 1.15e-05 | 1.27e-04 |
| 62) | TNF sR-I | P19438 | -0.538 | 1.15e-05 | 1.27e-04 |
| 63) | Angiostatin | P00747 | 0.538 | 1.15e-05 | 1.27e-04 |
| 64) | Gelsolin | P06396 | 0.538 | 1.15e-05 | 1.27e-04 |
| 65) | MAPK14 | Q16539 | -0.538 | 1.15e-05 | 1.27e-04 |
| 66) | PBEF | P43490 | -0.538 | 1.15e-05 | 1.27e-04 |
| 67) | Contactin-4 | Q8IWV2 | 0.538 | 1.15e-05 | 1.27e-04 |
| 68) | IGFBP-7 | Q16270 | 0.538 | 1.15e-05 | 1.27e-04 |
| 69) | PGRP-S | O75594 | -0.538 | 1.15e-05 | 1.27e-04 |
| 70) | FN1.3 | P02751 | 0.538 | 1.15e-05 | 1.27e-04 |
| 71) | Myeloperoxidase | P05164 | -0.513 | 3.54e-05 | 2.94e-04 |
| 72) | ROR1 | Q01973 | 0.513 | 3.54e-05 | 2.94e-04 |
| 73) | Cadherin-5 | P33151 | 0.513 | 3.54e-05 | 2.94e-04 |
| 74) | Carbonic anhydrase 6 | P23280 | 0.513 | 3.54e-05 | 2.94e-04 |
| 75) | FETUB | Q9UGM5 | 0.513 | 3.54e-05 | 2.94e-04 |
| 76) | FN1.4 | P02751 | 0.513 | 3.54e-05 | 2.94e-04 |
| 77) | DKK3 | Q9UBP4 | 0.513 | 3.54e-05 | 2.94e-04 |
| 78) | BPI | P17213 | -0.513 | 3.54e-05 | 2.94e-04 |
| 79) | Factor B | P00751 | -0.513 | 3.54e-05 | 2.94e-04 |
| 80) | Lysozyme | P61626 | -0.513 | 3.54e-05 | 2.94e-04 |
| 81) | bFGF-R | P11362 | 0.513 | 3.54e-05 | 2.94e-04 |
| 82) | Protein S | P07225 | -0.513 | 3.54e-05 | 2.94e-04 |
| 83) | Apo A-I | P02647 | 0.513 | 3.54e-05 | 2.94e-04 |
| 84) | MPIF-1 | P55773 | -0.513 | 3.54e-05 | 2.94e-04 |
| 85) | GDF-9 | O60383 | -0.513 | 3.54e-05 | 2.94e-04 |
| 86) | BGH3 | Q15582 | 0.513 | 3.54e-05 | 2.94e-04 |
| 87) | α1-Antitrypsin | P01009 | -0.513 | 3.54e-05 | 2.94e-04 |
| 88) | CYTD | P28325 | 0.513 | 3.54e-05 | 2.94e-04 |
| 89) | RACK1 | P63244 | 0.513 | 3.54e-05 | 2.94e-04 |
| 90) | C6 | P13671 | -0.513 | 3.54e-05 | 2.94e-04 |
| 91) | 14-3-3 eta | Q04917 | -0.513 | 3.54e-05 | 2.94e-04 |
| 92) | ITI heavy chain H4 | Q14624 | -0.513 | 3.54e-05 | 2.94e-04 |
| 93) | HRG | P04196 | 0.513 | 3.54e-05 | 2.94e-04 |
| 94) | SDF-1a | P48061 | 0.487 | 1.03e-04 | 6.84e-04 |
| 95) | SAP | P02743 | -0.487 | 1.03e-04 | 6.84e-04 |
| 96) | Thyroxine-Binding Globulin | P05543 | 0.487 | 1.03e-04 | 6.84e-04 |
| 97) | NAP-2 | P02775 | -0.487 | 1.03e-04 | 6.84e-04 |
| 98) | contactin-1 | Q12860 | 0.487 | 1.03e-04 | 6.84e-04 |
| 99) | TIG2 | Q99969 | -0.487 | 1.03e-04 | 6.84e-04 |
| 100) | CATC | P53634 | -0.487 | 1.03e-04 | 6.84e-04 |
| 101) | C2 | P06681 | -0.487 | 1.03e-04 | 6.84e-04 |
| 102) | CD109 | Q6YHK3 | 0.487 | 1.03e-04 | 6.84e-04 |
| 103) | Thrombin | P00734 | -0.487 | 1.03e-04 | 6.84e-04 |
| 104) | 14-3-3 protein gamma | P61981 | -0.487 | 1.03e-04 | 6.84e-04 |
| 105) | C3b | P01024 | -0.487 | 1.03e-04 | 6.84e-04 |
| 106) | CTAP-III | P02775 | -0.487 | 1.03e-04 | 6.84e-04 |
| 107) | C3d | P01024 | -0.487 | 1.03e-04 | 6.84e-04 |
| 108) | HGF | P14210 | -0.487 | 1.03e-04 | 6.84e-04 |
| 109) | Alkaline phosphatase, bone | P05186 | -0.487 | 1.03e-04 | 6.84e-04 |
| 110) | HAI-1 | O43278 | 0.487 | 1.03e-04 | 6.84e-04 |
| 111) | PAI-1 | P05121 | -0.487 | 1.03e-04 | 6.84e-04 |
| 112) | IP-10 | P02778 | -0.487 | 1.03e-04 | 6.84e-04 |
| 113) | AK1A1 | P14550 | -0.487 | 1.03e-04 | 6.84e-04 |
| 114) | ZAP70 | P43403 | -0.487 | 1.03e-04 | 6.84e-04 |
| 115) | VEGF121 | P15692 | -0.487 | 1.03e-04 | 6.84e-04 |
| 116) | Sonic Hedgehog | Q15465 | 0.487 | 1.03e-04 | 6.84e-04 |
